# The role of the ZEB1–neuroinflammation axis in CNS disorders

**DOI:** 10.1186/s12974-022-02636-2

**Published:** 2022-11-19

**Authors:** Elham Poonaki, Ulf Dietrich Kahlert, Sven G. Meuth, Ali Gorji

**Affiliations:** 1 Department of Neurology, Faculty of Medicine, Heinrich-Heine-University, Düsseldorf, Germany; 2 Epilepsy Research Center, Department of Neurosurgery, Westfälische Wilhelms-Universität Münster, Domagkstr. 11, 48149 Münster, Germany; 3 Molecular and Experimental Surgery, Faculty of Medicine, University Clinic for General-, Visceral-, Vascular- and Transplantation Surgery, Otto-Von-Guericke-University, Magdeburg, Germany; 4 Shefa Neuroscience Research Center, Khatam Alanbia Hospital, Tehran, Iran; 5 Neuroscience Research Center, Mashhad University of Medical Sciences, Mashhad, Iran

**Keywords:** Glioblastoma, Microglia, Metastasis, Neural stem cells, MicroRNA

## Abstract

Zinc finger E-box binding homeobox 1 (ZEB1) is a master modulator of the epithelial–mesenchymal transition (EMT), a process whereby epithelial cells undergo a series of molecular changes and express certain characteristics of mesenchymal cells. ZEB1, in association with other EMT transcription factors, promotes neuroinflammation through changes in the production of inflammatory mediators, the morphology and function of immune cells, and multiple signaling pathways that mediate the inflammatory response. The ZEB1–neuroinflammation axis plays a pivotal role in the pathogenesis of different CNS disorders, such as brain tumors, multiple sclerosis, cerebrovascular diseases, and neuropathic pain, by promoting tumor cell proliferation and invasiveness, formation of the hostile inflammatory micromilieu surrounding neuronal tissues, dysfunction of microglia and astrocytes, impairment of angiogenesis, and dysfunction of the blood–brain barrier. Future studies are needed to elucidate whether the ZEB1–neuroinflammation axis could serve as a diagnostic, prognostic, and/or therapeutic target for CNS disorders.

## Introduction

Central nervous system (CNS) disorders, such as multiple sclerosis (MS), Alzheimer’s disease (AD), neuropathic pain, and glioblastoma (GBM), are of high scientific interest worldwide due to their increasing prevalence and a lack of effective therapies [1, 2]. Focusing on metabolic pathways to identify relevant biomarkers involved in the pathogenesis of neuroinflammation in disease may contribute to the advent of novel diagnostic and therapeutic strategies [3–5]. Epithelial–mesenchymal transition (EMT) is a crucial biological process in which a cell loses epithelial characteristics, like cell–cell adhesion, and converts into motile non-polarized mesenchymal cells with invasive properties [6]. The conversion from epithelial to mesenchymal cells facilitates cell proliferation, migration, and invasion [7, 8]. EMT contributes to the pathophysiological mechanisms of wound healing, tissue fibrosis, and tumorigenesis [6]. Three different types of EMT have been distinguished: type I, which is observed during embryogenesis; type II, which occurs during wound healing and tissue fibrosis; and type III, which is activated during the spread of cancer cells [9]. The regulatory role of EMT on proliferating cells may be affected by various factors, such as inflammation, which contributes to the pathological processes of numerous neurological disorders [10, 11]. For example, alteration of transforming growth factor β (TGF-B) expression, the most potent activator of EMT, as well as genes involved in EMT may be of importance in the induction of chronic neuroinflammation in AD [12]. Moreover, EMT has been suggested to play a role in inflammation-related carcinogenesis [13, 14]. EMT is induced mainly through a series of EMT-promoting transcription factors (EMT-TFs), such as Twist-related protein 1 (TWIST1), Snail family proteins, and zinc finger E-box binding homeobox-1 (ZEB1) and -2 (ZEB2) [15]. The activation of EMT-TFs, such as ZEB1, is associated with loss of cellular connectivity and changes in epithelial apical–basal polarity, leading to changes in cell properties and alterations to their metabolic patterns [16].

The expression of ZEB1 differs markedly among various adult human tissues, showing low expression in the prostate, pancreas, and liver, moderate expression in the heart, mammary gland, and ovary, and high expression in the thymus, aorta, uterus, and bladder [5, 17]. ZEB1 and ZEB2 are involved in the regulation of uterine quiescence and contractility during pregnancy and labor [18]. Furthermore, ZEB1 modulates T-cell development in the thymus [19], controls self-renewal and generation of functional glandular structures in the prostate [20], governs cutaneous wound healing [21], regulates the activation of hepatic stellate cells [22], and maintains mammary basal cell fate and stem cell quiescence [23]. ZEB1 also plays a critical role in the development of embryos [17]. ZEB1 expression is essential for the maintenance of embryonic cells in undifferentiated states, as well as in the appropriate maturation and migration during development [24, 25]. Excessive expression of ZEB1, as a δ1-crystallin enhancer, has been observed in several organs of chicken embryos, including the nerve system, heart, thymus, lung, and lens [17]. ZEB1 regulates EMT late in gestation and is crucial for the capacity of embryos to develop into fetuses [26]. ZEB1 overexpression in a mouse model indicated lack of ZEB1 is associated with greater mortality during the perinatal period due to severe T-cell insufficiency, respiratory disorder, and skeletal deficiency [19]. Mutation of the *Zeb1* gene could have also resulted in a cleft palate as well as other craniofacial and skeletal anomalies [19]. A link between proliferative impairment in a subset of bone marrow-derived progenitors with *Zeb1* gene mutation has been suggested [20]. Moreover, evidence suggests the modulatory effects of Zeb1 on the differentiation of embryonic stem cells via the regulation of various cytoplasmic and nuclear proteins [24].

Alterations of ZEB1 expression regulate neural stem cell renewal and cell fate in the brain [27]. The expression of ZEB1 is pivotal for the balance between epithelial and mesenchymal gene expression as well as the proliferation of progenitor cells [28]. Loss of epithelial properties following ZEB1 activation is involved in several pathological conditions. ZEB1 alone or together with other EMT-TFs plays a key role in the metastasis of brain cancers [29–31]. Suppression of EMT by knocking down ZEB1 has been achieved in several studies [32–34], and inhibition of ZEB1 expression may prevent aggressive tumor progression [35]. Activation of ZEB1 can lead to chemoresistance in different types of cancers via the downregulation of E-cadherin [36, 37]. ZEB1 has an impact on pediatric solid tumors, such as neuroblastoma, via long intergenic noncoding RNAs (lncRNAs) and microRNAs (miRNAs) [38]. Moreover, ZEB1 contributes to the regulation of immune system development and function [39, 40]. Here, we summarize the current understanding of the regulatory mechanisms of ZEB1 in the CNS. Furthermore, we provide a comprehensive review of the current knowledge regarding the potential roles of ZEB1 in neurological disorders.

## ZEB1 structure

ZEB1, also known as TCF8 and δEF-1, belongs to the ZEB family encoded by the *ZEB1* gene on chromosome 10p11.2 [41, 42]. ZEB1 is a DNA-binding protein, which contains a homeodomain and two C2H2-type zinc finger clusters and binds to two high-affinity binding sites (E-boxes) [43]. Its DNA-binding activity is related to two zinc finger clusters present in the structure, which is essential for the recognition of the 5′-CANNTG 3′ sequence [36, 44, 45]. The ZEB1 protein is composed of 1117 amino acids with a homeodomain structure (HD) in the middle and a carboxy-terminal cluster [46]. The HD as the middle region is able to interact with the C-terminal binding protein (CtBP) and a Smad interaction domain [37]. ZEB1 proteins have Smad-interacting domains that modulate the TGF-β signal at the cell surface to affect gene regulation within the nucleus. The regulatory effect of ZEB1 is mediated via multiple motifs within the central area of this protein [47].

## Mechanism of ZEB1 and its effect on EMT

**Fig. 1:**
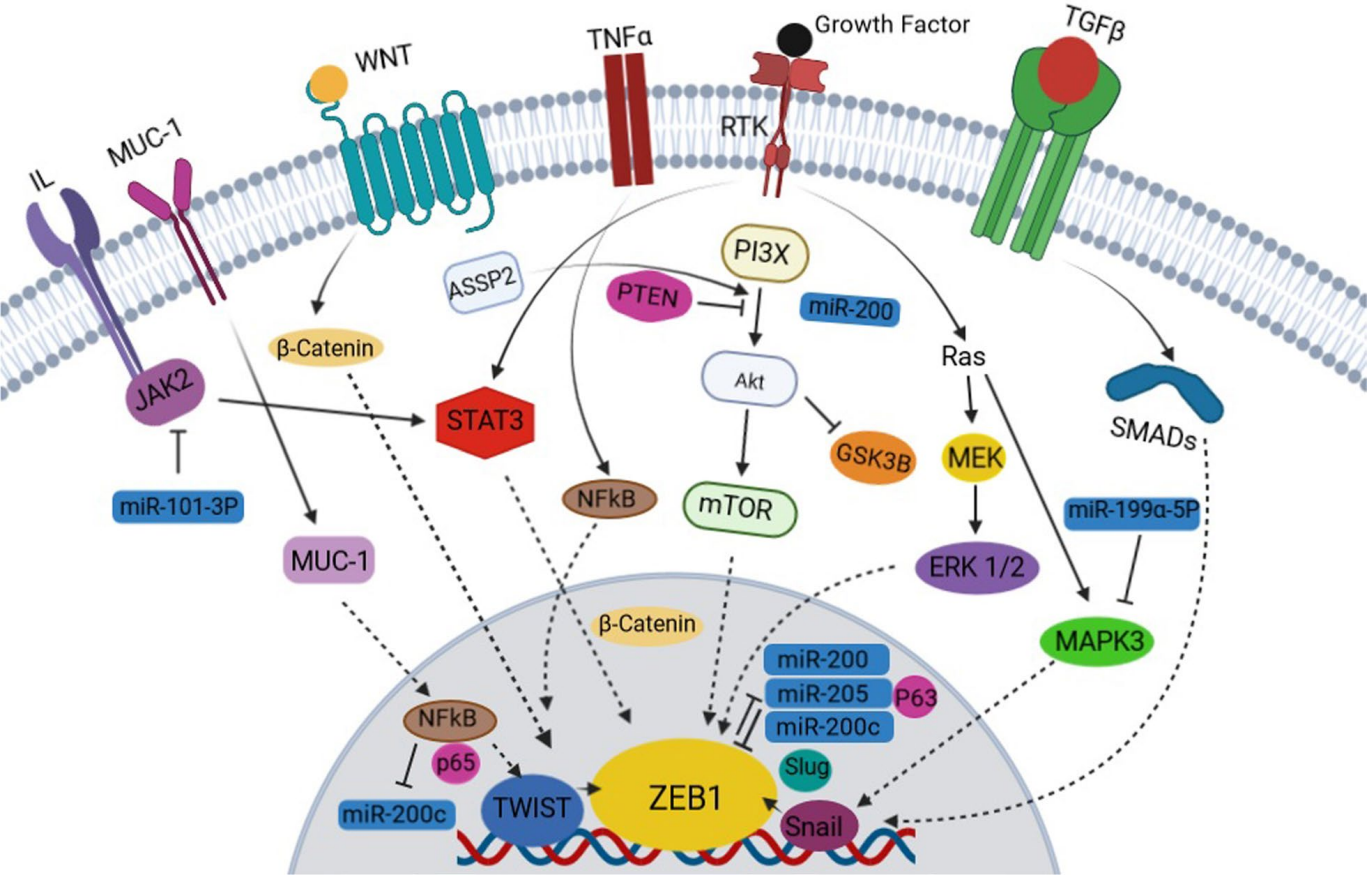
Zinc finger E-box binding homeobox 1 (ZEB1) represents a core transcriptional factor that controls essential intracellular processes. Upstream markers, including RAS/ERK, PI3X/AKT, NF-kB, JAK2/STAT3, Wnt/β-catenin, and Smad, lead to upregulation of ZEB1 via triggering ZEB1 directly or by mediation with TWIST, Snail and Slug. ZEB1 bilaterally is influenced via miR-200, miR200c, and miR-205. NF-kB also is involved in the upregulation of ZEB1 by repressing miR-200c. Transforming growth factor β (TGF-β), tumor necrosis factor (TNFα), Janus kinase 2(JAK2)

ZEB1 induces EMT by binding to the promoter region of the *E-cadherin* gene (*CDH1*) and preventing its expression. It is downstream of many cascades linked to EMT, such as TGF-β, nuclear factor kappa-light-chain-enhancer of activated B cells (NF-κB), Ras-Raf-MEK-ERK (RAS/ERK), Wnt/β-catenin, and tyrosine kinase receptors. Recent studies have focused on ZEB1 and its influence on EMT function causing both malignancy and chemotherapy resistance in tumors. In addition, this important transcription factor has influenced many CNS diseases through various pathways (Fig. 1).

## Effects of ZEB1 on the different signaling pathways

ZEB1 has been shown to be a major participant in various signaling pathways (Table 1). Expression of ZEB1 is modulated by multiple signaling pathways, like TGF-β, Wnt, NF-κB, hypoxia-inducible factor 1α (HIF-1α), cyclooxygenase-2 (COX-2), phosphatidylinositol 3-kinase (PI3K)/Ak strain transforming AKT (AKT) and AKT/mTOR as well as miRNAs [5, 47]. ZEB1 regulates the BMP/TGF-β pathway through its antagonist effect on TGF-β signaling. The TGF-β-mediated EMT effect on this pathway is induced via a stimulatory complex that binds the Smad domain along with binding to a co-activator consisting of P/CAF and P300 [48, 49]. TGF-β is a trigger of EMT together with signaling pathways such as Wnt, RAS, and Notch, similar to ZEB1 [50]. TGF-β acts as a potent marker for the activation of ZEB1 and Smad2, which promote EMT, tumorigenesis, and cancer recurrence [51]. However, increased levels of Smad2 and ZEB1 were also observed in cells that did not respond to TGF-β suppression, suggesting an alternative unknown mechanism [52, 53]. Finally, the significant interdependence between TGF-β and ZEB-1 and vice versa with miR200 has been shown to regulate EMT [54]. It has also been shown that the expression of ZEB1 can be affected by B-cell CLL/lymphoma 6 (BCL6), which stimulates transcriptional suppressors causing E-cadherin abrogation and EMT enhancement [55, 56].

**Table 1:** Various modulators of ZEB1 expression

Markers	ZEB1 suppressor	ZEB1 inducer	Mechanisms	Refs.
TGF-β		*	Making complex with SMADs and co-activators and involved in pathways (WNT, RAS, and Notch) to induce EMT	[22, 33, 80–85]
miR200c	*		Through ROS/miR-200c/ZEB1 axis suppresses ZEB1 and increase E-Cadherin	[60, 86–88]
ERK1/2		*	Directly activated ZEB1 via ERK/ZEB1 signaling pathway	[78, 79]
NF-kB		*	Activation of NF-kB signaling due to different markers such as IL-7 and MUC1 leads to ZEB1 induction	[22, 89]
AKT		*	It has an induction effect on ZEB1 through PI3K/AKT/mTOR	[66, 67, 69, 90, 91]
ASSP2		*	Correlated with the PI3K/AKT pathway	[71]
TKB1	*		Augmenting GSK-3B which is deterrence on radiation-induced EMT by repressing ZEB1	[64]
MiR-200	*		Works as a suppressor of ZEB1 and tumor invasion	[29, 54, 72, 92]
MUC1		*	Creating a complex with ZEB1 intermediating NF-κB p65 leads to miR-200c repression and contributes to EMT activation	[60]
Snail		*	Corporation with ZEB1 and TGFβ to increase tumor invasiveness	[63]
miR-33b	*		Inactivation of the Wnt/β-catenin/ZEB1 pathway concludes to EMT silencing	[76, 77]
PI3K		*	Involves in different pathways in the induction of EMT	[65–68, 70, 72, 90]
ESE1	*		Downregulating through the MEK–ERK pathway, resulted in overexpression of ZEB and EMT upregulation	[78]
miR-708	*		Suppressing ZEB1 resulted in EMT reduction	[68]
miR-199a-5p	*		Suppressing ZEB1 resulted in EMT reduction	[69]

AKT: Ak strain transforming, ASPP2: Apoptosis-stimulating protein of P53, EMT: Epithelial-mesenchymal transition, ESE1: Epithelial-specific ETS transcription factor 1, GSK-3B: Glycogen synthase kinase-3B, MUC1: Mucin 1, NF-κ β: Nuclear factor kappaβ, TGF-β: Transforming growth factor β, TKB1: Tyrosine kinase B1

Furthermore, ZEB1 causes EMT induction through the suppression of E-cadherin via the ctBP-independent pathway by linking to the SWI/SNF BRG1, a chromatin restructuring protein. E-cadherin expression increases by preventing linkage between ZEB1 and BRG1 [57]. CtBP has been identified as a critical co-repressor for the regulation of BCL6 [58, 59]. The mucin 1 (MUC1) forms a complex with ZEB1, which mediates NF-κB and p65, suppresses miR-200c, and contributes to EMT activation [60]. Anoikis (detachment-induced apoptosis) can be suppressed by receptor tyrosine kinase (TrKB) at the cell surface. The crucial role of ZEB1 in TrkB-induced EMT leads to the suppression of anoikis has also been demonstrated [61, 62]. Moreover, ZEB1 stimulates TrKB and promotes EMT by the modulation of a Twist–Snail axis [63]. Conversely, tyrosine kinase B1 (TKB1) increases glycogen synthase kinase-3β (GSK-3β), which prevents the induction of EMT, by suppressing ZEB1 [64]. Indeed, GSK-3β is the major factor of the Wnt/β-catenin cascade, and together with the phosphatidylinositol 3-kinase PI3K/AKT pathway, may control ZEB1 expression [65]. Serine/threonine kinase B also known as protein kinase B (PKB) or AKT is responsible for stimulating ZEB1 through the modulation of some signaling pathways, such as PI3K/AKT and/or AKT/mTOR [66, 67]. It has been shown that miR-708 and miR-199a-5p with a similar function are able to downregulate ZEB1 and reduce EMT through the PI3K/AKT/mTOR cascade [68, 69]. Aside from this, the apoptosis-stimulating protein of P53 (ASPP2), which correlates with the PI3K/AKT pathway, forms an intermediate complex with β-catenin and promotes EMT through ZEB1-mediated suppression of E-cadherin [70, 71]. ZEB1 enhances cell migration via the regulation of miR-200 and PI3K signaling [72]. ZEB1 modulates the polarization of M2-polarized tumor-associated macrophages (TAMs) via EMT regulation [73]. Furthermore, Zeb1 regulates T-cell migration [74]. FLF3, an antagonist of the oncogenic pathway and a negative regulator of EMT, inhibits ZEB1 transcription and regulates the Wnt and RAS oncogenic pathways [75]. MiR-33b suppresses ZEB1 which can lead to EMT silencing via inactivating the Wnt/β-catenin/ZEB1 signaling pathway [76, 77]. Epithelial-specific ETS transcription factor 1 (ESE1) is induced in the epithelial confined state and luminal subtype of breast cancer. ESE1 is downregulated by the MEK–ERK pathway, leading to overexpression of ZEB1 and upregulation of EMT [78]. The ERK1/2-mediated signaling cascades ERK/ZEB1 axis stimulates EMT and regulates cell apoptosis and migration [79].

## Effects of ZEB1 on various cell types of the CNS

ZEB1 plays a crucial role in cell differentiation and migration as well as cell fate in the CNS. The function of ZEB1 has a complex transcriptional regulatory effect on neural progenitor cells. It can act as a repressive marker in embryonic NSC proliferation and migration via interaction with CTBP2 and the regulation of Neurod1 and Pard6b [93]. Conversely, it plays a key role in the differentiation of human embryonic stem cells into neurons [94]. The HIF-1α pathway regulates neuronal polarization, maturation, and differentiation through the modulation of ZEB1 values [95]. Furthermore, Zeb1 affects the trans-differentiation of mouse embryonic fibroblasts into functional neurons [96]. In the absence of ZEB1, immature differentiation and mal-migration of neurons and radial glial cells occur due to the activity of Pak3, a p21-activated serine/threonine-protein protein kinase [97]. Moreover, ZEB1 is necessary for the differentiation of radial glia-like stem cells, which are required in the adult hippocampus for the formation of neurons and astrocytes [27]. ZEB1 also controls the onset of astrocyte precursor emigration from the ventricular zone and regulates the timing of their differentiation via the modulation of the adhesion protein Cadherin-1 [98]. Moreover, ZEB1 expression in neurons may act as a regulator of their differentiation by repressing polarity genes in neural stem cells [99]. ZEB1 is also a prerequisite factor for NSC migration [100]. It has been suggested that ZEB1 is implicated in the regulation of retinal organoid development [101].

## Malfunctions of ZEB1

ZEB1 is implicated in the pathological mechanisms of various diseases. Mutations in *ZEB1* in humans have been linked to multiple developmental malformations, such as posterior polymorphous corneal dystrophy with corpus callosum maldevelopment, malformations of the inner ear, obesity, and cleft palate [102, 103]. In addition to the initiation of corneal cell apoptosis, stromal fibrosis, and squamous metaplasia, the loss of function of ZEB1 inhibits corneal vascularization and activates immune-mediated processes of the ocular surface [104]. Furthermore, it has been suggested that tumor cells that have undergone EMT obtain stem cell properties including, self-renewal, invasiveness, radioresistance, and chemoresistance [5]. ZEB1 plays a critical role in the regulation of DNA damage by controlling EMT in multiple tissues [44] and modulates cancer cell differentiation and invasiveness, vascular functionality, tumor angiogenesis, and immune responses [105]. It has been elucidated that increased ZEB1 activity influenced by hyaluronic acid through the ZEB1/epithelial splicing regulatory protein 1/CD44 axis promotes EMT and tumor invasion in breast cancer [106]. BCL6 also has a positive effect on the induction of EMT associated with the increase of ZEB1, leading to the suppression of E-cadherin and consequently tumor progression [56]. Silencing ZEB1 also decreases PD-L1 as an immune checkpoint ligand along with the downregulation of miR200 and consequently EMT activations in cancer cells [92]. Ultrasound-targeted microbubble destruction, a novel therapeutic approach, has been suggested as a means of inhibiting cell migration via the suppression of ZEB1 and deactivation of EMT by targeting the ROS/miR-200c/ZEB1 axis [107]. Moreover, miR-200c suppressed ZEB1 via the modulation of the PI3K/Akt pathway and the function of TGFβ in non-small cell lung cancer [33, 90]. The activation of mir200a is linked to the suppression of the Wnt/β-catenin pathway that leads to overexpression of E-cadherin [108]. Downregulation of ZEB1 also affects the enhancement of apoptosis of cancer cells, followed by a reduction in tumor invasiveness and migration through the expression of Wnt5a and vimentin [109]. The key role of HIF-1α in promoting EMT through the activation of ZEB1 is substantial tumorigenicity [110, 111]. Furthermore, the negative effect of the circadian gene timeless, an essential protein that modulates circadian rhythm, on ZEB1 overexpression and EMT values has been described [112]. CSN5 is an oncogenic marker that directly interacts with ZEB1 and enhances its stability while promoting EMT in renal cell carcinoma cells [113]. Assessment of the function of X-inactive specific transcript (XIST) revealed that it mainly represses miR-429, a tumor suppressor, and then results in a higher expression of ZEB1 and EMT via the critical axis of XIST/miR-429/ZEB1 and enhances tumor cell invasiveness [114]. MiR-127 also has an attenuating effect on cell proliferation by targeting ZEB1 on smooth muscle cells [115]. The inhibitory function of miR-200c on ZEB1 is also elucidated in trastuzumab-resistant gastric cancer, leading to suppression of EMT and enhancement of drug sensitivity [116]. The activity of NF-κB has been demonstrated to stimulate the induction of ZEB1 and EMT through the action of interleukin-17 (IL-17) and phosphorylation of ezrin Tyr353, respectively, in different cancers [91, 117]. ZEB1 serves an important role in controlling the size of the neural progenitor pool, neuronal migration, and cleavage plane orientation of dividing progenitor cells. Upon the knockout of *Zeb1*, an extra number of premature neurons are produced and the cleavage plane of mitotic progenitor cells fails to orientate appropriately, resulting in random orientation and premature neuronal differentiation, particularly in the upper layer of neocortical tissues. It has been suggested that a malfunction of ZEB1 together with its effector Pak3 could contribute to neocortical developmental disorders [97].

## ZEB1 contributes to inflammatory responses

Several studies have demonstrated the critical role of ZEB1 in promoting inflammatory responses. ZEB1 is crucial for the development of both T-cell and B-cell development [39]. Mutations of ZEB1 have been implicated in T-cell immunodeficiency [89]. ZEB1 is a pivotal element to maintain immune cell viability, mobility, and cytokine expression, and regulates the expression of various cytokines, such as IL-1b, IL-6, IL-8, and TNF-α, by the modulation of the TGF-β-related Stat3 signaling pathways and Nf-κb [22, 80–82, 118–120]. Conversely, several pro-inflammatory cytokines, like TGF-β, increase the expression of ZEB1 via the activation of Smad, TK receptors, NF-κB, and the JAK1–STAT3 signaling pathways [121].

**Fig. 2:**
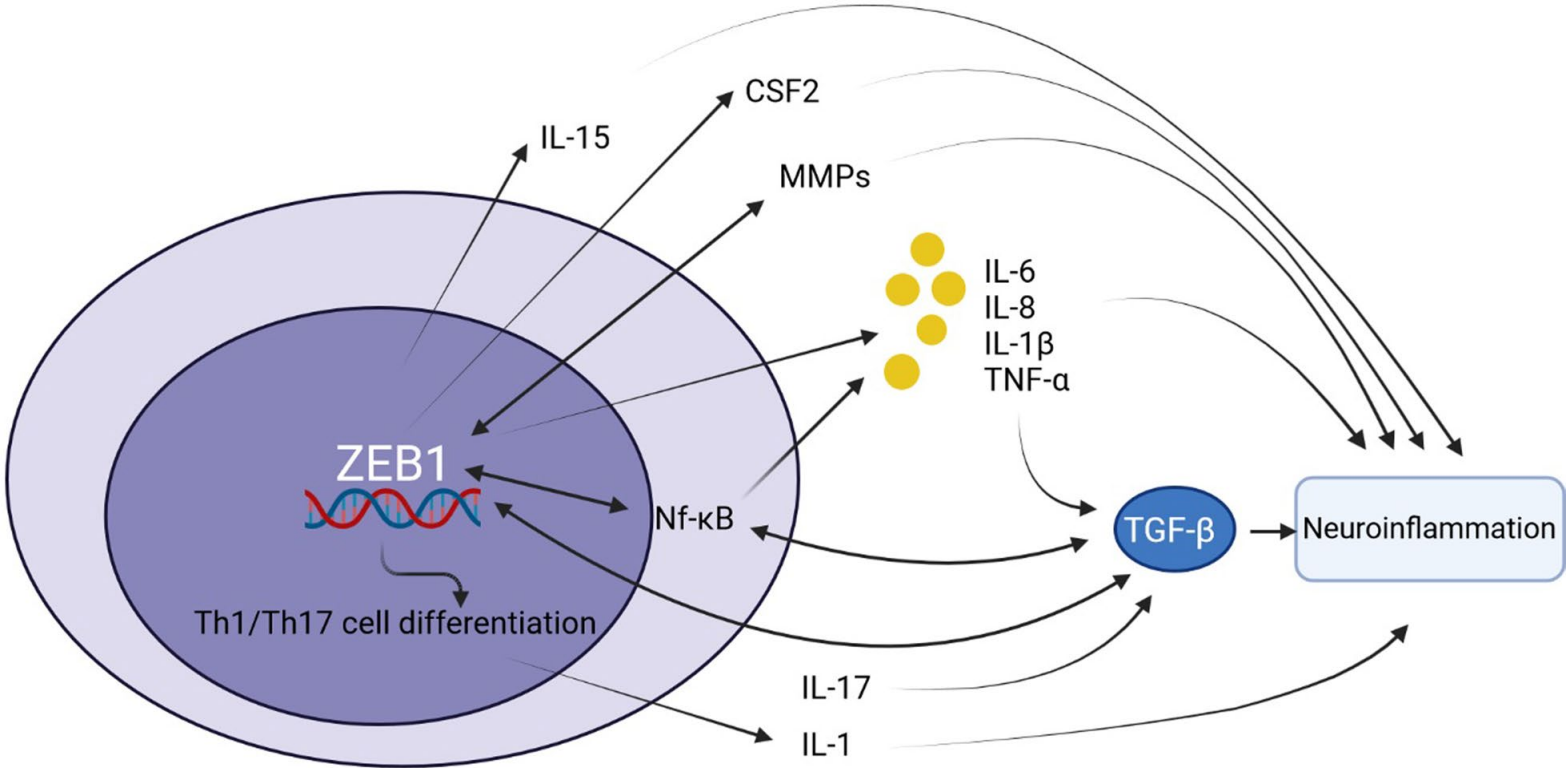
The schematic diagram shows the role of ZEB1 interaction with various inflammatory mediators in the induction of neuroinflammation. Interleukin (IL), matrix metalloproteinases (MMPs), nuclear factor kappa B (NF-κB), Type 1 T helper: Th1, T helper 17 cells: Th17, colony-stimulating factor 2 (CSF2), tumor necrosis factor-α (TNF-α), transforming growth factor-β (TGF-β)

IL-1β upregulates ZEB1 expression and promotes inflammation [86]. ZEB1 modulates the expression of several inflammatory response genes. Direct enhancement of the production of inflammatory cytokines, such as IL-6 and IL-8, initiates inflammatory processes and facilitates tumor growth [122]. ZEB1-mediated immune responses also contribute to inflammation in the tumor micromilieu via its direct regulatory effect on the expression of IL-6 [123]. ZEB1 induction of programmed death-ligand 1 and CD47 contributes to the formation of the hostile inflammatory microenvironment surrounding tumors [74]. ZEB1 promotes inflammatory responses through the suppression of *N*-methyl purine glycosylase, a DNA glycosylase, in epithelial cells via the induction of inflammatory mediators, such as IL-1β, and the generation of reactive oxygen species [124]. Furthermore, IL-17 influences the upregulation of the ZEB1-mediated NF-κB pathway as well as tumor cell migration through stimulation of EMT [117]. Sodium tanshinone IIA sulfonate, an antioxidant and anti-inflammatory substance, can prevent EMT by targeting ZEB1, Snail1, and the Smad signaling pathway [125]. Using lipopolysaccharide application to induce local inflammation in the lungs enhances tumor cell migration through a Zeb1-dependent mechanism [126]. The enhancement of TNF-α values increases ZEB1 as a target of miR-200c and miR-141 in cells, suppresses E-cadherin, and regulates EMT progression [87]. MiR-9 directly targets NF-κB that leads to inflammatory responses in lymphatic endothelial cells via the promotion of EMT-associated genes, such as ZEB1 (Fig. 2) [87].

Microglia act as the first line of the innate immune defence [127], and astrocytes are pivotal regulators of both innate and adaptive immune responses in the CNS [128]. The expression of ZEB1 in both microglia and astrocyte critically contribute to neuroinflammation in the CNS [129]. The expression of ZEB1 regulates microglia immune responses to CNS insults and reduces the production of astrocytic CXCL1 via the TGFβ-dependent signaling pathway [11]. ZEB1 enhances the neuronal output of neural stem cells of the hippocampus at the expense of glial cells [27]. The *ZEB1* gene has been reported to be involved in cognitive impairment in humans [83]. The reduction in Zeb1/2 and lncRNA-1604 in the neocortex and striatum can lead to a neurodegenerative process in a mouse model of Huntington’s disease [130]. The dysregulation of the lncRNA-1604/miR-200c/ZEB axis during neural differentiation could also lead to neurodegenerative diseases [131]. Fused in sarcoma, an RNA-binding protein linked to neurodegenerative diseases acts through miR-200c and its target transcript ZEB1 [132]. Loss of ubiquilin 1, a protein critical for combating neurological disorders linked to protein aggregation, significantly increases the expression of ZEB1 [133].

## ZEB1 in CNS disorders

**Fig. 3:**
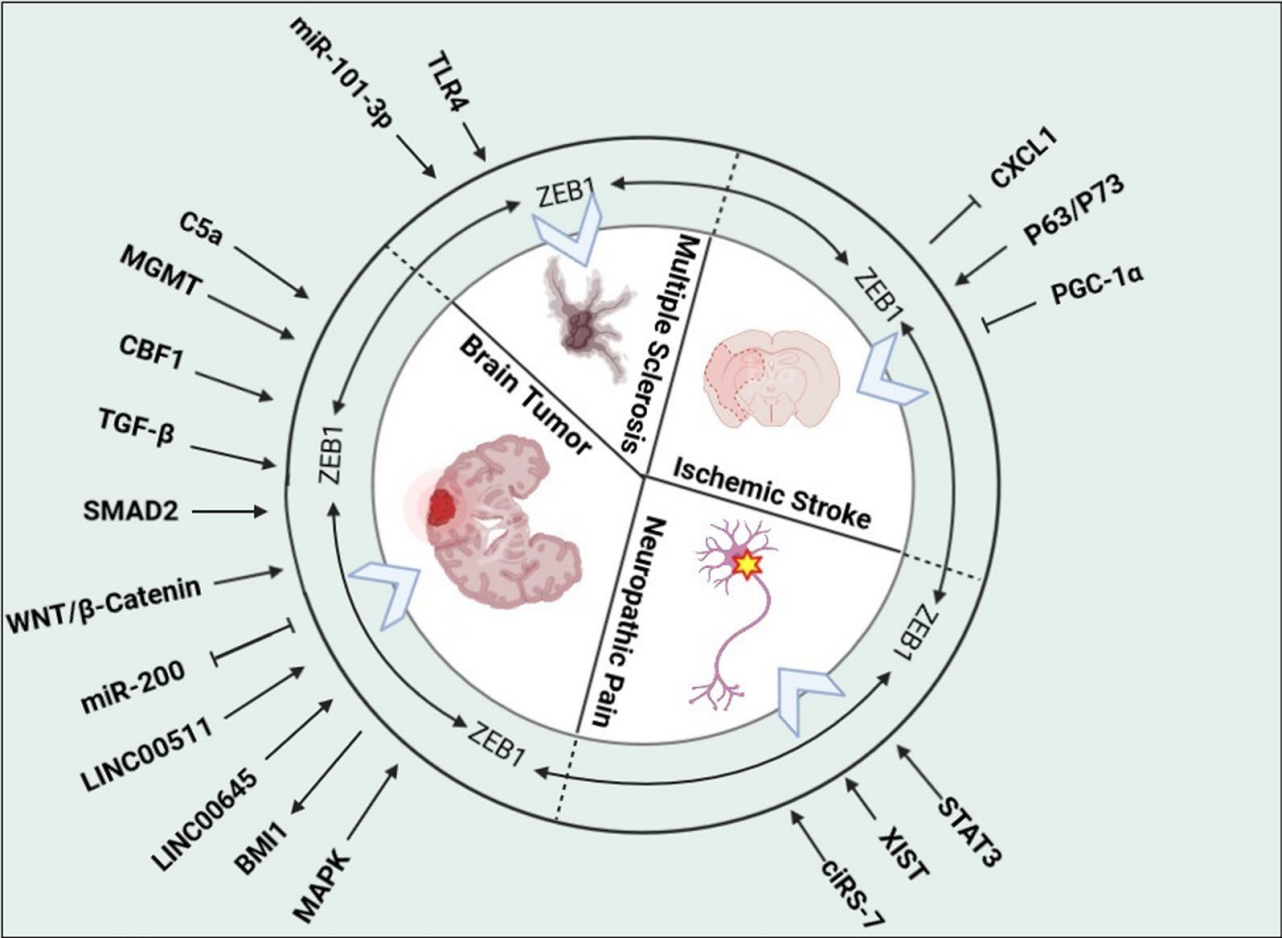
The schematic diagram shows the relationship between ZEB1 and its downstream signaling pathways with various CNS disorders

Several studies have shown that ZEB1, along with other factors such as TWIST, Snail, and Slug, is an important transcription factor involved in EMT promotion in the CNS and plays an important role in the pathophysiology of different neurological disorders, including brain tumors, neuropathic pain, acute ischemic stroke, and MS (Fig. 3; Table 2).

**Table 2:** A summary of markers and signaling pathways and their related mechanisms implicated in ZEB1-related CNS disorders

CNS diseases	Markers and pathways	Mechanisms	Refs.
Cerebrovascular diseases	TGF-b1	Inhibition of astrocytic CXCL1 via TGF-b1 pathways	[83, 129]
	PGC-1α	Its expression happens after ischemic stroke to reduce brain damage via the downregulation of inflammatory cytokines and interaction with ZEB1	[134]
	p63, p73	ZEB1 creates a linkage between p63 and p73 for promoting the cell survival pathway	[135]
	CXCL1, TGF-β1 pathway	Brain protection by ZEB1 via CXCL1 inhibition in the TGF-β1 pathway	[129]
Neuropathic pain	XIST	Sponging miR-150 leads to overexpression of ZEB1 and neuropathic pain	[136]
	ciRS-7, STAT3	Sponging of miR-641 or activation of miR-135a-5p contributes to expressing pro-inflammatory cytokines, such as IL-6, IL-12, and TNFα leading to EMT induction	[137, 138]
GBM	MGMT	Lower effect of temozolomide therapy by the interference of ZEB1 with MGMT	[139]
	CBF1	Activating EMT inducers such as *ZEB1, SNAIL1, and CD44* genes	[140, 141]
	miR-205-3p, LINC00645, TGF-β	LINC00645 in collaboration with miR-205-3p and ZEB1 promotes EMT stimulated by TGF-β	[84]
	LINC00511, miR-524-5p,YB1	By influencing on ZEB1 promoted GBM aggression	[142]
	miRNA-200	GBM invasiveness increases followed by inhibition of miRNA-200 by ZEB1	[29]
	TGF-β, SMAD2	Along with ZEB1 lead to GBM cell aggression	[52]
	WNT/β-catenin, twist, snail	Wnt/β-catenin pathway via activation of EMT inducers such as ZEB1, twist, and Snail promotes GBM aggregation and tumor malignancy	[143]
	α6-integrin, FGFR1, YAP1, FOXM1	α6-integrin is involved in radioresistance as well as GBM stemness and proliferation by administering FGFR1 under the coordination of ZEB1 and YAP1 leading to FOXM1 stimulation	[144, 145]
	ma, MAPK	These markers with influence on ZEB1 expression lead to tumor invasion	[146]
Multiple sclerosis	JAK2, miR-101-3p	JAK2 is suppressed followed by the inhibition of miR-101-3p through ZEB1 activities and increasing cytokines relevant to pathogenicity	[147]
	*Zfhep1*, *Zfhep2*, *IL2*	IL2 was associated with T-cell formation and can be suppressed via ZEB1 (related to upregulation of *Zfhep1* and not *Zfhep2)*	[148, 149]
	TLR4, miR-200a-3p		[150, 151]
CNS traumatic injuries	ErbB2, TGF- β		[72, 152]
	XIST, miR-27a, Smurf1	Suppressing XIST caused to inhibit of miR-494 in the PTEN/AKT/mTOR signaling pathway leading to alleviating neuroinflammation via the deactivation of ZEB1 after SCI	[136, 153, 154]

## ZEB1 effects in CNS tumors

ZEB1 as a key element of EMT promotion has been extensively studied in different brain tumors, particularly GBM. GBM is the most aggressive malignant primary brain tumor in which a variety of therapeutic strategies have failed to demonstrate efficacy [155]. Several investigations have reported that the ZEB1 pathway contributes to GBM initiation and progression, invasion, radioresistance, and chemoresistance. ZEB1 is expressed in the tumor invasive zone of human GBM tissues, which is associated with hypoxic regions of the tumor [156]. The clinical studies demonstrate that GBM patients with a high level of *ZEB1/YAP1* gene signature have a shorter median overall survival [144]. The expression of Smad interacting protein 1 (SIP1, also known as ZEB2), a member of the ZEB group of transcription factors, plays a role in the impairment of colony formation and invasion of tumorigenic glioma cells through the regulation of E-cadherin and mesenchymal proteins, such as fibronectin and vimentin [157]. SIP1 and N-cadherin are also implicated in the migration of IL-1β/TGF-β-induced neurosphere cells from the human LN-229 glioma cell line [158, 159]. TGF-β showed a regulatory effect on ZEB1 expression along with Smad2 and EMT signaling leading to GBM cell aggression [52]. Moreover, the Wnt/β-catenin pathway promotes GBM aggregation and tumor malignancy via activation of EMT inducers such as ZEB1, Twist, and Snail [143]. Lef1, an effector of the Wnt signaling pathway, activates ZEB1 and enhances GBM cell migration and chemoresistance [160]. It has been revealed that GBM invasiveness is mediated by an alteration in N-cadherin dynamics, through the regulatory effect of ZEB1 on roundabout guidance protein 1 [139]. *BMI-1* is an important gene for controlling the proliferation and self-renewal of GBM cancer stem cells. Suppression of BMI1 has been shown to downregulate GBM stem cell proliferation [161]. It has also been shown that increasing ZEB1 in parallel with BMI1 can synergistically induce EMT and stem cell proliferation [162]. The EMT-activator ZEB1 is a promoter of metastasis, and SOX2 and BMI1 are targets of EMT activators, especially ZEB1 [29]. Mesenchymal stem-like cells expressed C5a contribute to ZEB1 expression and brain tumor invasiveness through the stimulation of p38 mitogen-activated protein kinase [146]. The interaction between ZEB1 and O-6-methylguanine-DNA methyltransferase, the most reliable prognostic marker for GBM therapy resistance, has been demonstrated in poor response to temozolomide (TMZ) through the upregulation of c-MYB by the ZEB1–miR-200 feedback loop [139]. Furthermore, ZEB1 was a crucial role in TMZ resistance in GBM cells through the upregulation of c-MYB by the ZEB1–miR-200 pathway [139]. The antagonistic effect of IL-24 on ZEB1 leads to a suppression of GBM cell migration and invasion as well as an enhancement of the chemosensitivity of tumor cells to TMZ [163]. Alfa-6-integrin, a regulator of GBM proliferation and stemness, regulates the expression of GBM stem cells via the modulation of the ZEB1/YAP1 transcription complex that leads to enhancement of cell proliferation and stemness via the stimulation of the forkhead box M1 gene [144] and promotes radioresistance of GBM cells through the modulation of DNA damage response [145]. The E3 ubiquitin-protein ligase parkinsonian protein 2 acts as a ZEB1 antagonist, inhibits EMT, and prevents GBM cell invasion [164]. Hypoxia is a strong inducer of a mesenchymal shift in GBM cells, which is associated with increased migration and invasiveness of GBM cells. Knockdown of HIF1α has been shown to not only suppress ZEB1, but also inhibit the mesenchymal trans-differentiation in GBM [100, 156]. Moreover, the direct effect of recombination signal binding protein for immunoglobulin kappa J on the activation of EMT inducers, such as *ZEB1, SNAIL1, and CD44* genes, could increase the invasiveness of GBM cells, which has been correlated with the hypoxic pseudopalisading regions [140, 141]. The function of lncRNAs has been studied in various cancers, including GBM [165, 166]. LINC00645, a lncRNA, was identified as a collaborator with miR-205-3p and ZEB1 in a signaling pathway promoting EMT stimulated by TGF-β. Indeed, miR-205-3p modulated TGF-β under the function of LINC00645, leading to the invasiveness of gliomas [84]. Moreover, LINC00511 in conjunction with the LINC00511/miR-524-5p/YB1/ZEB1 axis contributes to EMT. While LINC00511 is influenced by ZEB1, it indirectly upregulates YB1 via sponging miR-524-5p and promotes GBM tumorigenesis [142]. Moreover, the lncRNA ZEB1 antisense 1 (ZEB1-AS1) showed excessive expression in cancer cells, such as gliomas, compared with primary normal cells [167]. ZEB1-AS1 enhanced tumorigenesis and proliferation of GBM cells by interacting with miR200c/141 [168]. The interaction between miR-200c and miR-141 decreased EMT as well as glioma cell growth and aggression by downregulating ZEB1 [88, 168]. A link between isocitrate dehydrogenase 1 (IDH1) and ZEB1 expression has been reported in lower-grade glioma. The expression of ZEB1 was enhanced in IDH1/2-mutant gliomas, and IDH1/2-mutant gliomas exhibited significantly lower values of ZEB1 protein [169]. The role of ZEB1 as a potential prognosis biomarker and drug target in medulloblastoma has been reported [170].

Various miRNAs have shown therapeutic potential by acting on GBM cancer cell migration and invasion via the modulation of ZEB1. GBM invasion increases through the inhibition of the ZEB1–miRNA-200 axis effects on stem cell markers, such as OLIG2, SOX2, and CD133 [29]. miR-200c negatively modulates ZEB1 regulation and consequently reduces the migration of these cells [171]. Similarly, miR-574 independently targeted ZEB1 in GBM cells, which contributed to the inhibition of GBM proliferation [172]. miR-205 also inhibited the proliferation of GBM cells by affecting the Akt/mTOR cascade via interaction with ZEB1 [173]. Moreover, metformin suppresses the AKT/mTOR/ZEB1 signaling pathway via the inhibition of the TGF-β1-induced EMT-like process in GBM cancer stem cells [174]. Moreover, the activation of miR-590-3p, as well as miR-139-5p, prevented GBM tumor invasion by targeting ZEB1 and ZEB2 and inhibiting EMT [175, 176].

## ZEB1 and MS

Although the exact role of ZEB1 in MS needs to be clarified, some studies have clarified the different roles of ZEB1 in the pathology of MS. The dysfunction of brain endothelial cells plays a role in the initiation of neuroinflammation and cell injury in MS. A link between the damage of the blood–brain barrier (BBB) and the EMT process has been suggested [177]. Evidence suggests the implication of ZEB1 in the dysfunction of BBB under pathological conditions [178]. Furthermore, one of the hallmarks of MS is inappropriate activation of interferon-gamma (IFN-γ)-producing Th1 and Th17 cells [179]. ZEB1 contributes to pathogenic Th1 and Th17 cell differentiation in MS. ZEB1 mutation inhibits the expression of miR-101-3p, which leads to the suppression of the Janus kinase/signal transducer and activator of transcription (JAK/STAT) pathway and excessive release of IL-17 and IFN-γ [180]. Dysregulation of the JAK/STAT pathway plays an important role in the pathophysiology of several autoimmune diseases, including MS [181]. Moreover, ZEB1 regulates IL-2 expression and is implicated in T-cell development through the modulation of the balance between Zfhep1 and not Zfhep2, the splice variants of ZEB1, in an experimental model of MS [147–149]. Knockdown of ZEB1 in dendritic cells decreases IL-12 secretion and increases Th2 differentiation [182]. The highly upregulated liver cancer (HULC), an lncRNA determined to be upregulated in patients with MS, may be involved in MS progression. HULC activates the miR-200a-3p/ZEB1 signaling pathway and regulates EMT [150].

## ZEB1 and neuropathic pain

ZEB1-related neuroinflammatory responses could contribute to neuropathic pain. The miR-28-5p/ZEB1 pathway has been suggested as a potential therapeutic target for neuropathic pain. Overexpression of miR-28-5p reduces neuropathic pain behaviors in a chronic sciatic nerve injury model in rats through the inhibition of neuroinflammation induced by the release of Cox-2, IL-6, and IL-1β. MiR-28-5p binds to the 3-untranslated area of Zeb1, downregulates *Zeb1* expression, and inhibits cytokine expression [183]. Moreover, miR-128-3p and miR-96-5p are reported to suppress *Zeb1* expression and regulate neuroinflammation and neuropathic pain [184, 185]. In addition, miR-200b/miR-429 serves as a key regulator of neuropathic pain via targeting ZEB1 [186]. lncRNA X-inactive specific transcript (XIST) increased significantly in the spinal cord tissues and microglia in a chronic constriction injury rat model. Silencing ZEB1 alleviated neuropathic pain and downregulated the expression of XIST through the regulatory effects of miR-150 [136]. Circular RNAs, such as ciRS-7, increased EMT by upregulating ZEB1 and STAT3. This was associated with either sponging of miR-641 or activation of miR-135a-5p, which contributed to the expression of pro-inflammatory cytokines, like IL-6, IL-12, and TNFα, and the induction of neuropathic pain [137, 138]. Furthermore, lncRNAs, such as LINC00657, contribute to the development of neuropathic pain in animal models of chronic pain through the regulation of the miR-136/ZEB1 axis. MiR-136 regulates neuroinflammatory responses as well as the expression of ZEB1. Inhibition of ZEB1 inhibits neuropathic pain behaviors in vivo [187]. Oxaliplatin-induced chronic pain enhanced the values of ZEB1 in the spinal dorsal horn neurons through the regulatory effects of NF-κB and/or Ras/Erk as well as by triggering the interaction between DNA (cytosine-5)-methyltransferase-3β and ZEB1 [188].

## ZEB1 and ischemic/traumatic brain injuries

The induction of ZEB1 is part of a neuroprotective response by neurons after brain ischemic insults. High values of ZEB1 expression in neocortical tissues are observed in neocortical specimens of patients with stroke [135]. ZEB1 regulates microglial activities in acute ischemic stroke. After the induction of brain ischemia, ZEB1 expression significantly increases in the ischemic cerebral tissues, particularly in microglia. A greater ramified morphology of microglia in ischemic tissues is associated with a higher expression of ZEB1, which enables microglia to react more precisely to stimuli and promotes inflammatory responses following ischemic events. Furthermore, in an experimental ischemic stroke model, upregulation of ZEB1 leads to the inhibition of astrocytic CXCL1 expression during the response to TGF-β1-dependent signaling and reduction in the entrance of neutrophils into the brain. It has been suggested that targeting ZEB1 expression may lead to moderate acute ischemic brain injuries [83, 129]. The expression of PPARγ-coactivator-1α (PGC-1α) enhances after ischemic stroke and reduces brain damage by downregulating neuroinflammatory cytokines and regulating neurotrophins. ZEB1 expression was indirectly related to PGC-1α through the modulation of Sirt1 [134]. ZEB1 expression promotes cell survival in the neocortex after acute ischemic insults through the modulation of proapoptotic isoforms of p63 and p73 [135]. In addition, the application of ZEB1 antagomir improved neurological function and cerebral edema, and decreased the expression of TNF-α, IL-1β, IL-6, and GFAP in ischemic tissues in an intracerebral hemorrhage rat model [189].

The astrocytic response to CNS injury is implicated in EMT and upregulation in ZEB expression. CNS injury-related astrogliosis enhances EMT and its related gene expression. In experimental models of spinal cord injury or transient ischemic stroke, the knockdown of the *Zeb2f* in astrocytes lessened astrogliosis, induced greater lesions, and delayed functional motor recovery [190]. Following CNS injury, meningeal cells actively migrate into the injury site undergoing EMT and build the meningeal barrier between normal and injured tissues, which is regulated by the TGF-β1/non-Smad/SNAI1 pathway [191]. Expression values of the TGF-β receptor and the Ephrins receptor (ErbB2) are greatly enhanced in the meningeal cells of the injury site to maintain the integrity and homeostasis of CNS cells within the lesion site [152]. Thus, ZEB1 could be a potential target to regulate tissue reconstruction after CNS injuries. The suppression of XIST after spinal cord injury (SCI) enhances the function of the XIST/miR-27a/Smurf1 pathway and causes the inhibition of miR-494. This process leads to the alleviation of neuroinflammation via the deactivation of ZEB1 and the reduction of neuroinflammatory mediators, such as COX-2, TNF-α, and IL-6 [136, 153, 154].

## Conclusion

Considering the critical role of neuroinflammation in various diseases, EMT is known to be an important process of pathogenicity in various neurological disorders. ZEB1 as a focal transcription factor of EMT has been considered a potential target for the prognosis and treatment of various neurological diseases. Although recognition of the exact role of ZEB1 in CNS dysfunction requires additional analysis and evaluation, it was concluded that ZEB1, in partnership with other EMT transcription factors, has dominant functions in EMT and neuroinflammation. The complex interaction between ZEB1, immune cells, and various cytokines is tightly connected to neuroinflammation through the regulation of different signaling pathways. The ZEB1–inflammation axis plays a crucial role in the pathogenesis of various CNS disorders through promoting tumor cell proliferation and invasiveness, formation of the hostile inflammatory micromilieu surrounding neuronal tissues, dysfunction of BBB, dysfunction of microglia and astrocytes, and disturbances of angiogenesis. The molecular mechanisms and signaling pathways orchestrating the association between ZEB1 and inflammatory processes are linked with the initiation, progression, and outcomes of various CNS disorders. Future studies are required to demonstrate whether the modulation of ZEB1 could play a beneficial role as a diagnostic, prognostic, and/or therapeutic approach for CNS disorders.

## Data Availability

Not applicable.

## Abbreviations

AKT: Ak strain transforming
AD: Alzheimer’s disease
ASPP2: Apoptosis-stimulating protein of P53
BCL6: B-cell CLL/lymphoma 6
EMT: Epithelial–mesenchymal transition
CtBP: C-terminal binding protein
CNS: Central nervous system
COX-2: Cyclooxygenase-2
CSF2: Colony-stimulating factor 2
EMT-TFs: EMT-promoting transcription factors
ESE1: Epithelial-specific ETS transcription factor 1
GBM: Glioblastoma
GSK-3B: Glycogen synthase kinase-3B
HD: Homeodomain structure
IFN-γ: Interferon-gamma
lncRNAs: Long intergenic noncoding RNAs
HIF-1α: Hypoxia-inducible factor 1α
IL: Interleukin
IDH1: Isocitrate dehydrogenase 1
JAK2: Janus kinase 2
TNFα: Tumor necrosis factor
MMPs: Matrix metalloproteinases
MS: Multiple sclerosis
NF-κ β: Nuclear factor kappaβ
MUC1: Mucin 1
PGC-1α: PPARγ-coactivator-1α
PKB: Protein kinase B
PI3K: Phosphatidylinositol 3-kinase
Th1: T helper 1
Th17: T helper 17
TMZ: Temozolomide
TGF-β: Transforming growth factor β
TKB1: Tyrosine kinase B1
TWIST1: Twist-related protein 1
XIST: X-inactive specific transcript
ZEB1: Zinc finger E-box binding homeobox 1

## References

[1] Ingusci S, Verlengia G, Soukupova M, Zucchini S, Simonato M (2019). Gene therapy tools for brain diseases. Front Pharmacol.

[2] Ingle AP, Shende S, Gupta I, Nagaonkar D, Pandit R, Paralikar P, Rai M (2018). Metal nanoparticles in management of diseases of the central nervous system. The microbiology of central nervous system infections.

[3] Kaddurah-Daouk R, Krishnan KR (2009). Metabolomics: a global biochemical approach to the study of central nervous system diseases. Neuropsychopharmacology.

[4] Stephenson J, Nutma E, van der Valk P, Amor S (2018). Inflammation in CNS neurodegenerative diseases. Immunology.

[5] Zhang P, Sun Y, Ma L (2015). ZEB1: at the crossroads of epithelial–mesenchymal transition, metastasis and therapy resistance. Cell Cycle.

[6] Serrano-Gomez SJ, Maziveyi M, Alahari SK (2016). Regulation of epithelial–mesenchymal transition through epigenetic and post-translational modifications. Mol Cancer.

[7] Kalluri R (2009). EMT: when epithelial cells decide to become mesenchymal-like cells. J Clin Investig.

[8] Yilmaz M, Christofori G (2009). EMT, the cytoskeleton, and cancer cell invasion. Cancer Metastasis Rev.

[9] Ashrafizadeh M, Ang HL, Moghadam ER, Mohammadi S, Zarrin V, Hushmandi K, Samarghandian S, Zarrabi A, Najafi M, Mohammadinejad R, Kumar AP (2020). MicroRNAs and their influence on the ZEB family: mechanistic aspects and therapeutic applications in cancer therapy. Biomolecules.

[10] Kang D-H (2020). Loosening of the mesothelial barrier as an early therapeutic target to preserve peritoneal function in peritoneal dialysis. Kidney Res Clin Pract.

[11] Ricciardi M, Zanotto M, Malpeli G, Bassi G, Perbellini O, Chilosi M, Bifari F, Krampera M (2015). Epithelial-to-mesenchymal transition (EMT) induced by inflammatory priming elicits mesenchymal stromal cell-like immune-modulatory properties in cancer cells. Br J Cancer.

[12] Imran SA, Yazid MD, Idrus RBH, Maarof M, Nordin A, Razali RA, Lokanathan Y (2021). Is there an interconnection between epithelial–mesenchymal transition (EMT) and telomere shortening in aging?. Int J Mol Sci.

[13] Zhou C, Liu J, Tang Y, Liang X (2012). Inflammation linking EMT and cancer stem cells. Oral Oncol.

[14] Lopez-Novoa JM, Nieto MA (2009). Inflammation and EMT: an alliance towards organ fibrosis and cancer progression. EMBO Mol Med.

[15] Feldker N, Ferrazzi F, Schuhwerk H, Widholz SA, Guenther K, Frisch I, Jakob K, Kleemann J, Riegel D, Bönisch U (2020). Genome-wide cooperation of EMT transcription factor ZEB 1 with YAP and AP-1 in breast cancer. EMBO J.

[16] Stemmler MP, Eccles RL, Brabletz S, Brabletz T (2019). Non-redundant functions of EMT transcription factors. Nat Cell Biol.

[17] Hurt EM, Saykally JN, Anose BM, Kalli KR, Sanders MM (2008). Expression of the ZEB1 (deltaEF1) transcription factor in human: additional insights. Mol Cell Biochem.

[18] Renthal NE, Chen CC, Williams KC, Gerard RD, Prange-Kiel J, Mendelson CR (2010). miR-200 family and targets, ZEB1 and ZEB2, modulate uterine quiescence and contractility during pregnancy and labor. Proc Natl Acad Sci USA.

[19] Almotiri A, Alzahrani H, Menendez-Gonzalez JB, Abdelfattah A, Alotaibi B, Saleh L, Greene A, Georgiou M, Gibbs A, Alsayari A, Taha S, Thomas LA, Shah D, Edkins S, Giles P, Stemmler MP, Brabletz S, Brabletz T, Boyd AS, Siebzehnrubl FA, Rodrigues NP (2021). Zeb1 modulates hematopoietic stem cell fates required for suppressing acute myeloid leukemia. J Clin Invest.

[20] Wang X, Xu H, Cheng C, Ji Z, Zhao H, Sheng Y, Li X, Wang J, Shu Y, He Y, Fan L, Dong B, Xue W, Wai Chua C, Wu D, Gao WQ, He Zhu H (2020). Identification of a Zeb1 expressing basal stem cell subpopulation in the prostate. Nat Commun.

[21] Singh K, Sinha M, Pal D, Tabasum S, Gnyawali SC, Khona D, Sarkar S, Mohanty SK, Soto-Gonzalez F, Khanna S, Roy S, Sen CK (2019). Cutaneous epithelial to mesenchymal transition activator ZEB1 regulates wound angiogenesis and closure in a glycemic status-dependent manner. Diabetes.

[22] Li L-Y, Yang C-C, Yang J-F, Li H-D, Zhang B-Y, Zhou H, Hu S, Wang K, Huang C, Meng X-M (2019). ZEB1 regulates the activation of hepatic stellate cells through Wnt/β-catenin signaling pathway. Eur J Pharmacol.

[23] Han Y, Villarreal-Ponce A, Gutierrez G, Nguyen Q, Sun P, Wu T, Sui B, Berx G, Brabletz T, Kessenbrock K (2022). Coordinate control of basal epithelial cell fate and stem cell maintenance by core EMT transcription factor Zeb1. Cell Rep.

[24] Chen T, Pan P, Wei W, Zhang Y, Cui G, Yu Z, Guo X (2022). Expression of Zeb1 in the differentiation of mouse embryonic stem cell. Open Life Sci.

[25] Haensel D, Sun P, MacLean AL, Ma X, Zhou Y, Stemmler MP, Brabletz S, Berx G, Plikus MV, Nie Q, Brabletz T, Dai X (2019). An Ovol2-Zeb1 transcriptional circuit regulates epithelial directional migration and proliferation. EMBO Rep.

[26] Tada T, Takagi N, Adler I-D (1993). Parental imprinting on the mouse X chromosome: effects on the early development of X0, XXY and XXX embryos. Genet Res.

[27] Gupta B, Errington AC, Jimenez-Pascual A, Eftychidis V, Brabletz S, Stemmler MP, Brabletz T, Petrik D, Siebzehnrubl FA (2021). The transcription factor ZEB1 regulates stem cell self-renewal and cell fate in the adult hippocampus. Cell Rep.

[28] Liu Y, El-Naggar S, Darling DS, Higashi Y, Dean DC (2008). Zeb1 links epithelial–mesenchymal transition and cellular senescence. Development.

[29] Wellner U, Schubert J, Burk UC, Schmalhofer O, Zhu F, Sonntag A, Waldvogel B, Vannier C, Darling D, zur Hausen A, Brunton VG, Morton J, Sansom O, Schuler J, Stemmler MP, Herzberger C, Hopt U, Keck T, Brabletz S, Brabletz T (2009). The EMT-activator ZEB1 promotes tumorigenicity by repressing stemness-inhibiting microRNAs. Nat Cell Biol.

[30] Krebs AM, Mitschke J, LasierraLosada M, Schmalhofer O, Boerries M, Busch H, Boettcher M, Mougiakakos D, Reichardt W, Bronsert P (2017). The EMT-activator Zeb1 is a key factor for cell plasticity and promotes metastasis in pancreatic cancer. Nat Cell Biol.

[31] Díaz-López A, Díaz-Martín J, Moreno-Bueno G, Cuevas EP, Santos V, Olmeda D, Portillo F, Palacios J, Cano A (2015). Zeb1 and S nail1 engage mi R-200f transcriptional and epigenetic regulation during EMT. Int J Cancer.

[32] Takeyama Y, Sato M, Horio M, Hase T, Yoshida K, Yokoyama T, Nakashima H, Hashimoto N, Sekido Y, Gazdar AF, Minna JD, Kondo M, Hasegawa Y (2010). Knockdown of ZEB1, a master epithelial-to-mesenchymal transition (EMT) gene, suppresses anchorage-independent cell growth of lung cancer cells. Cancer Lett.

[33] Argast GM, Krueger JS, Thomson S, Sujka-Kwok I, Carey K, Silva S, O’Connor M, Mercado P, Mulford IJ, Young GD (2011). Inducible expression of TGFβ, snail and Zeb1 recapitulates EMT in vitro and in vivo in a NSCLC model. Clin Exp Metastasis.

[34] Ren J, Chen Y, Song H, Chen L, Wang R (2013). Inhibition of ZEB1 reverses EMT and chemoresistance in docetaxel-resistant human lung adenocarcinoma cell line. J Cell Biochem.

[35] Zhang Y, Liu G, Wu S, Jiang F, Xie J, Wang Y (2016). Zinc finger E-box-binding homeobox 1: its clinical significance and functional role in human thyroid cancer. Onco Targets Ther.

[36] Chava S, Gayatri MB, Reddy AB (2019). EMT contributes to chemoresistance in pancreatic cancer. Breaking tolerance to pancreatic cancer unresponsiveness to chemotherapy.

[37] Zhang Y, Xu L, Li A, Han X (2019). The roles of ZEB1 in tumorigenic progression and epigenetic modifications. Biomed Pharmacother.

[38] Fratini L, Jaeger M, de Farias CB, Brunetto AT, Brunetto AL, Shaw L, Roesler R (2021). Oncogenic functions of ZEB1 in pediatric solid cancers: interplays with microRNAs and long noncoding RNAs. Mol Cell Biochem.

[39] Arnold CN, Pirie E, Dosenovic P, McInerney GM, Xia Y, Wang N, Li X, Siggs OM, Karlsson Hedestam GB, Beutler B (2012). A forward genetic screen reveals roles for Nfkbid, Zeb1, and Ruvbl2 in humoral immunity. Proc Natl Acad Sci USA.

[40] Robert C, Tsiampali J, Fraser-Miller SJ, Neumann S, Maciaczyk D, Young SL, Maciaczyk J, Gordon KC (2021). Molecular monitoring of glioblastoma’s immunogenicity using a combination of Raman spectroscopy and chemometrics. Spectrochim Acta Part A Mol Biomol Spectrosc.

[41] Williams TM, Montoya G, Wu Y, Eddy RL, Byers MG, Shows TB (1992). The TCF8 gene encoding a zinc finger protein (Nil-2-a) resides on human chromosome 10p11.2. Genomics.

[42] Madany M, Thomas T, Edwards LA (2018). The curious case of ZEB1. Discoveries.

[43] Fontemaggi G, Gurtner A, Damalas A, Costanzo A, Higashi Y, Sacchi A, Strano S, Piaggio G, Blandino G (2005). deltaEF1 repressor controls selectively p53 family members during differentiation. Oncogene.

[44] Drapela S, Bouchal J, Jolly MK, Culig Z, Soucek K (2020). ZEB1: a critical regulator of cell plasticity, DNA damage response, and therapy resistance. Front Mol Biosci.

[45] Comijn J, Berx G, Vermassen P, Verschueren K, van Grunsven L, Bruyneel E, Mareel M, Huylebroeck D, van Roy F (2001). The two-handed E box binding zinc finger protein SIP1 downregulates E-cadherin and induces invasion. Mol Cell.

[46] Perez-Oquendo M, Gibbons DL (2022). Regulation of ZEB1 function and molecular associations in tumor progression and metastasis. Cancers.

[47] Brabletz S, Bajdak K, Meidhof S, Burk U, Niedermann G, Firat E, Wellner U, Dimmler A, Faller G, Schubert J, Brabletz T (2011). The ZEB1/miR-200 feedback loop controls Notch signalling in cancer cells. EMBO J.

[48] Postigo AA, Depp JL, Taylor JJ, Kroll KL (2003). Regulation of Smad signaling through a differential recruitment of coactivators and corepressors by ZEB proteins. EMBO J.

[49] Van Grunsven LA, Taelman V, Michiels C, Opdecamp K, Huylebroeck D, Bellefroid EJ (2006). δEF1 and SIP1 are differentially expressed and have overlapping activities during *Xenopus* embryogenesis. Dev Dyn Off Publ Am Assoc Anat.

[50] Fuxe J, Vincent T, Garcia de Herreros A (2010). Transcriptional crosstalk between TGFβ and stem cell pathways in tumor cell invasion: role of EMT promoting Smad complexes. Cell Cycle.

[51] David CJ, Huang Y-H, Chen M, Su J, Zou Y, Bardeesy N, Iacobuzio-Donahue CA, Massagué J (2016). TGF-β tumor suppression through a lethal EMT. Cell.

[52] Joseph JV, Conroy S, Tomar T, Eggens-Meijer E, Bhat K, Copray S, Walenkamp AM, Boddeke E, Balasubramanyian V, Wagemakers M (2014). TGF-β is an inducer of ZEB1-dependent mesenchymal transdifferentiation in glioblastoma that is associated with tumor invasion. Cell Death Dis.

[53] Hua W, Ten Dijke P, Kostidis S, Giera M, Hornsveld M (2020). TGFβ-induced metabolic reprogramming during epithelial-to-mesenchymal transition in cancer. Cell Mol Life Sci.

[54] Gregory PA, Bracken CP, Smith E, Bert AG, Wright JA, Roslan S, Morris M, Wyatt L, Farshid G, Lim YY, Lindeman GJ, Shannon MF, Drew PA, Khew-Goodall Y, Goodall GJ (2011). An autocrine TGF-beta/ZEB/miR-200 signaling network regulates establishment and maintenance of epithelial–mesenchymal transition. Mol Biol Cell.

[55] Li S, Zhang HY, Du ZX, Li C, An MX, Zong ZH, Liu BQ, Wang HQ (2016). Induction of epithelial–mesenchymal transition (EMT) by Beclin 1 knockdown via posttranscriptional upregulation of ZEB1 in thyroid cancer cells. Oncotarget.

[56] Yu JM, Sun W, Hua F, Xie J, Lin H, Zhou DD, Hu ZW (2015). BCL6 induces EMT by promoting the ZEB1-mediated transcription repression of E-cadherin in breast cancer cells. Cancer Lett.

[57] Sanchez-Tillo E, Lazaro A, Torrent R, Cuatrecasas M, Vaquero EC, Castells A, Engel P, Postigo A (2010). ZEB1 represses E-cadherin and induces an EMT by recruiting the SWI/SNF chromatin-remodeling protein BRG1. Oncogene.

[58] Mendez LM, Polo JM, Yu JJ, Krupski M, Ding BB, Melnick A, Ye BH (2008). CtBP is an essential corepressor for BCL6 autoregulation. Mol Cell Biol.

[59] Papadopoulou V, Postigo A, Sánchez-Tilló E, Porter AC, Wagner SD (2010). ZEB1 and CtBP form a repressive complex at a distal promoter element of the BCL6 locus. Biochem J.

[60] Rajabi H, Alam M, Takahashi H, Kharbanda A, Guha M, Ahmad R, Kufe D (2014). MUC1-C oncoprotein activates the ZEB1/miR-200c regulatory loop and epithelial–mesenchymal transition. Oncogene.

[61] Smit M, Peeper D (2011). Zeb1 is required for TrkB-induced epithelial–mesenchymal transition, anoikis resistance and metastasis. Oncogene.

[62] Kupferman M, Jiffar T, El-Naggar A, Yilmaz T, Zhou G, Xie T, Feng L, Wang J, Holsinger F, Yu D (2010). TrkB induces EMT and has a key role in invasion of head and neck squamous cell carcinoma. Oncogene.

[63] Smit MA, Geiger TR, Song JY, Gitelman I, Peeper DS (2009). A Twist-Snail axis critical for TrkB-induced epithelial–mesenchymal transition-like transformation, anoikis resistance, and metastasis. Mol Cell Biol.

[64] Liu W, Huang Y-J, Liu C, Yang Y-Y, Liu H, Cui J-G, Cheng Y, Gao F, Cai J-M, Li B-L (2014). Inhibition of TBK1 attenuates radiation-induced epithelial–mesenchymal transition of A549 human lung cancer cells via activation of GSK-3β and repression of ZEB1. Lab Invest.

[65] Wu K, Fan J, Zhang L, Ning Z, Zeng J, Zhou J, Li L, Chen Y, Zhang T, Wang X (2012). PI3K/Akt to GSK3β/β-catenin signaling cascade coordinates cell colonization for bladder cancer bone metastasis through regulating ZEB1 transcription. Cell Signal.

[66] Chen W, Wu S, Zhang G, Wang W, Shi Y (2013). Effect of AKT inhibition on epithelial–mesenchymal transition and ZEB1-potentiated radiotherapy in nasopharyngeal carcinoma. Oncol Lett.

[67] Zhao L, Li X, Song N, Li A, Hou K, Qu X, Che X, Liu Y (2018). Src promotes EGF-induced epithelial-to-mesenchymal transition and migration in gastric cancer cells by upregulating ZEB1 and ZEB2 through AKT. Cell Biol Int.

[68] Sun S, Hang T, Zhang B, Zhu L, Wu Y, Lv X, Huang Q, Yao H (2019). miRNA-708 functions as a tumor suppressor in colorectal cancer by targeting ZEB1 through Akt/mTOR signaling pathway. Am J Transl Res.

[69] Liu Y, Lu C, Fan L, Wang J, Li T, Liu Z, Sheng J, Qian R, Duan A, Lu D (2020). MiR-199a-5p targets ZEB1 to inhibit the epithelial–mesenchymal transition of ovarian ectopic endometrial stromal cells via PI3K/Akt/mTOR signal pathway in vitro and in vivo. Reprod Sci.

[70] Wu T, Song H, Xie D, Zhao B, Xu H, Wu C, Hua K, Deng Y, Ji C, Hu J, Fang L (2018). Silencing of ASPP2 promotes the proliferation, migration and invasion of triple-negative breast cancer cells via the PI3K/AKT pathway. Int J Oncol.

[71] Wang Y, Bu F, Royer C, Serres S, Larkin JR, Soto MS, Sibson NR, Salter V, Fritzsche F, Turnquist C (2014). ASPP2 controls epithelial plasticity and inhibits metastasis through β-catenin-dependent regulation of ZEB1. Nat Cell Biol.

[72] Yang Y, Ahn YH, Chen Y, Tan X, Guo L, Gibbons DL, Ungewiss C, Peng DH, Liu X, Lin SH, Thilaganathan N, Wistuba II, Rodriguez-Canales J, McLendon G, Creighton CJ, Kurie JM (2014). ZEB1 sensitizes lung adenocarcinoma to metastasis suppression by PI3K antagonism. J Clin Invest.

[73] Cortés M, Sanchez-Moral L, de Barrios O, Fernández-Aceñero MJ, Martínez-Campanario M, Esteve-Codina A, Darling DS, Győrffy B, Lawrence T, Dean DC (2017). Tumor-associated macrophages (TAMs) depend on ZEB1 for their cancer-promoting roles. EMBO J.

[74] Guo Y, Lu X, Chen Y, Rendon B, Mitchell RA, Cuatrecasas M, Cortes M, Postigo A, Liu Y, Dean DC (2021). Zeb1 induces immune checkpoints to form an immunosuppressive envelope around invading cancer cells. Sci Adv.

[75] Liu D, Skomorovska Y, Song J, Bowler E, Harris R, Ravasz M, Bai S, Ayati M, Tamai K, Koyuturk M (2019). ELF3 is an antagonist of oncogenic-signalling-induced expression of EMT-TF ZEB1. Cancer Biol Ther.

[76] Zhang P, Bai H, Liu G, Wang H, Chen F, Zhang B, Zeng P, Wu C, Peng C, Huang C, Song Y, Song E (2015). MicroRNA-33b, upregulated by EF24, a curcumin analog, suppresses the epithelial-to-mesenchymal transition (EMT) and migratory potential of melanoma cells by targeting HMGA2. Toxicol Lett.

[77] Qu J, Li M, An J, Zhao B, Zhong W, Gu Q, Cao L, Yang H, Hu C (2015). MicroRNA-33b inhibits lung adenocarcinoma cell growth, invasion, and epithelial–mesenchymal transition by suppressing Wnt/beta-catenin/ZEB1 signaling. Int J Oncol.

[78] Sinh ND, Endo K, Miyazawa K, Saitoh M (2017). Ets1 and ESE1 reciprocally regulate expression of ZEB1/ZEB2, dependent on ERK1/2 activity, in breast cancer cells. Cancer Sci.

[79] Chiu L, Hsin I, Yang T, Sung W, Chi J, Chang J, Ko J, Sheu G (2017). The ERK–ZEB1 pathway mediates epithelial–mesenchymal transition in pemetrexed resistant lung cancer cells with suppression by vinca alkaloids. Oncogene.

[80] Chen L, He X, Xie Y, Huang Y, Wolff DW, Abel PW, Tu Y (2018). Up-regulated miR-133a orchestrates epithelial–mesenchymal transition of airway epithelial cells. Sci Rep.

[81] Vandewalle C, Van Roy F, Berx G (2009). The role of the ZEB family of transcription factors in development and disease. Cell Mol Life Sci.

[82] Kim TH, Rowat AC, Sloan EK (2016). Neural regulation of cancer: from mechanobiology to inflammation. Clin Transl Immunol.

[83] Chen Q, Zhang F, Qu K, Hanif Q, Shen J, Jia P, Ning Q, Zhan J, Zhang J, Chen N, Chen H, Huang B, Lei C (2020). Genome-wide association study identifies genomic loci associated with flight reaction in cattle. J Anim Breed Genet.

[84] Li C, Zheng H, Hou W, Bao H, Xiong J, Che W, Gu Y, Sun H, Liang P (2019). Long non-coding RNA linc00645 promotes TGF-β-induced epithelial–mesenchymal transition by regulating miR-205-3p-ZEB1 axis in glioma. Cell Death Dis.

[85] Urena-Peralta JR, Alfonso-Loeches S, Cuesta-Diaz CM, Garcia-Garcia F, Guerri C (2018). Deep sequencing and miRNA profiles in alcohol-induced neuroinflammation and the TLR4 response in mice cerebral cortex. Sci Rep.

[86] Dohadwala M, Wang G, Heinrich E, Luo J, Lau O, Shih H, Munaim Q, Lee G, Hong L, Lai C, Abemayor E, Fishbein MC, Elashoff DA, Dubinett SM, St John MA (2010). The role of ZEB1 in the inflammation-induced promotion of EMT in HNSCC. Otolaryngol Head Neck Surg.

[87] Chakraborty S, Zawieja DC, Davis MJ, Muthuchamy M (2015). MicroRNA signature of inflamed lymphatic endothelium and role of miR-9 in lymphangiogenesis and inflammation. Am J Physiol Cell Physiol.

[88] Guo E, Wang Z, Wang S (2016). MiR-200c and miR-141 inhibit ZEB1 synergistically and suppress glioma cell growth and migration. Eur Rev Med Pharmacol Sci.

[89] Dean KC, Huang L, Chen Y, Lu X, Liu Y (2015). An Rb1-dependent amplification loop between Ets1 and Zeb1 is evident in thymocyte differentiation and invasive lung adenocarcinoma. BMC Mol Biol.

[90] Zhou G, Zhang F, Guo Y, Huang J, Xie Y, Yue S, Chen M, Jiang H, Li M (2017). miR-200c enhances sensitivity of drug-resistant non-small cell lung cancer to gefitinib by suppression of PI3K/Akt signaling pathway and inhibits cell migration via targeting ZEB1. Biomed Pharmacother.

[91] Wang Y, Lin Z, Sun L, Fan S, Huang Z, Zhang D, Yang Z, Li J, Chen W (2014). Akt/Ezrin Tyr353/NF-κB pathway regulates EGF-induced EMT and metastasis in tongue squamous cell carcinoma. Br J Cancer.

[92] Noman MZ, Janji B, Abdou A, Hasmim M, Terry S, Tan TZ, Mami-Chouaib F, Thiery JP, Chouaib S (2017). The immune checkpoint ligand PD-L1 is upregulated in EMT-activated human breast cancer cells by a mechanism involving ZEB-1 and miR-200. Oncoimmunology.

[93] Wang H, Xiao Z, Zheng J, Wu J, Hu XL, Yang X, Shen Q (2019). ZEB1 represses neural differentiation and cooperates with CTBP2 to dynamically regulate cell migration during neocortex development. Cell Rep.

[94] Jiang Y, Yan L, Xia L, Lu X, Zhu W, Ding D, Du M, Zhang D, Wang H, Hu B (2018). Zinc finger E-box-binding homeobox 1 (ZEB1) is required for neural differentiation of human embryonic stem cells. J Biol Chem.

[95] Kullmann JA, Trivedi N, Howell D, Laumonnerie C, Nguyen V, Banerjee SS, Stabley DR, Shirinifard A, Rowitch DH, Solecki DJ (2020). Oxygen tension and the VHL-Hif1α pathway determine onset of neuronal polarization and cerebellar germinal zone exit. Neuron.

[96] Yan L, Li Y, Shi Z, Lu X, Ma J, Hu B, Jiao J, Wang H (2017). The zinc finger E-box-binding homeobox 1 (Zeb1) promotes the conversion of mouse fibroblasts into functional neurons. J Biol Chem.

[97] Liu J, Liu Y, Shao J, Li Y, Qin L, Shen H, Xie Y, Xia W, Gao W-Q (2019). Zeb1 is important for proper cleavage plane orientation of dividing progenitors and neuronal migration in the mouse neocortex. Cell Death Differ.

[98] Ohayon D, Garces A, Joly W, Soukkarieh C, Takagi T, Sabourin JC, Agius E, Darling DS, De Santa Barbara P, Higashi Y, Stolt CC, Hugnot JP, Richardson WD, Carroll P, Pattyn A (2016). Onset of spinal cord astrocyte precursor emigration from the ventricular zone involves the Zeb1 transcription factor. Cell Rep.

[99] Singh S, Howell D, Trivedi N, Kessler K, Ong T, Rosmaninho P, Raposo AA, Robinson G, Roussel MF, Castro DS, Solecki DJ (2016). Zeb1 controls neuron differentiation and germinal zone exit by a mesenchymal–epithelial-like transition. Elife.

[100] Kahlert UD, Suwala AK, Raabe EH, Siebzehnrubl FA, Suarez MJ, Orr BA, Bar EE, Maciaczyk J, Eberhart CG (2015). ZEB1 promotes invasion in human fetal neural stem cells and hypoxic glioma neurospheres. Brain Pathol.

[101] Godini R, Fallahi H (2022). Dynamics of transcription regulatory network during mice-derived retina organoid development. Gene.

[102] Kurima K, Hertzano R, Gavrilova O, Monahan K, Shpargel KB, Nadaraja G, Kawashima Y, Lee KY, Ito T, Higashi Y, Eisenman DJ, Strome SE, Griffith AJ (2011). A noncoding point mutation of Zeb1 causes multiple developmental malformations and obesity in Twirler mice. PLoS Genet.

[103] Jang MS, Roldan AN, Frausto RF, Aldave AJ (2014). Posterior polymorphous corneal dystrophy 3 is associated with agenesis and hypoplasia of the corpus callosum. Vis Res.

[104] Zhang Y, Liu X, Liang W, Dean DC, Zhang L, Liu Y (2021). Expression and function of ZEB1 in the cornea. Cells.

[105] Fu R, Li Y, Jiang N, Ren BX, Zang CZ, Liu LJ, Lv WC, Li HM, Weiss S, Li ZY, Lu T, Wu ZQ (2020). Inactivation of endothelial ZEB1 impedes tumor progression and sensitizes tumors to conventional therapies. J Clin Invest.

[106] Preca BT, Bajdak K, Mock K, Lehmann W, Sundararajan V, Bronsert P, Matzge-Ogi A, Orian-Rousseau V, Brabletz S, Brabletz T, Maurer J, Stemmler MP (2017). A novel ZEB1/HAS2 positive feedback loop promotes EMT in breast cancer. Oncotarget.

[107] Shi D, Guo L, Sun X, Shang M, Meng D, Zhou X, Liu X, Zhao Y, Li J (2020). UTMD inhibit EMT of breast cancer through the ROS/miR-200c/ZEB1 axis. Sci Rep.

[108] Cong N, Du P, Zhang A, Shen F, Su J, Pu P, Wang T, Zjang J, Kang C, Zhang Q (2013). Downregulated microRNA-200a promotes EMT and tumor growth through the wnt/β-catenin pathway by targeting the E-cadherin repressors ZEB1/ZEB2 in gastric adenocarcinoma. Oncol Rep.

[109] Xue Y, Zhang L, Zhu Y, Ke X, Wang Q, Min H (2019). Regulation of proliferation and epithelial-to-mesenchymal transition (EMT) of gastric cancer by ZEB1 via modulating Wnt5a and related mechanisms. Med Sci Monit.

[110] Zhang W, Shi X, Peng Y, Wu M, Zhang P, Xie R, Wu Y, Yan Q, Liu S, Wang J (2015). HIF-1α promotes epithelial–mesenchymal transition and metastasis through direct regulation of ZEB1 in colorectal cancer. PLoS ONE.

[111] Zhu J, Huang Z, Zhang M, Wang W, Liang H, Zeng J, Wu K, Wang X, Hsieh JT, Guo P (2018). HIF-1α promotes ZEB1 expression and EMT in a human bladder cancer lung metastasis animal model Corrigendum in/10.3892/ol.2021.12860. Oncol Lett.

[112] Colangelo T, Carbone A, Mazzarelli F, Cuttano R, Dama E, Nittoli T, Albanesi J, Barisciano G, Forte N, Palumbo O, Graziano P, di Masi A, Colantuoni V, Sabatino L, Bianchi F, Mazzoccoli G (2022). Loss of circadian gene timeless induces EMT and tumor progression in colorectal cancer via Zeb1-dependent mechanism. Cell Death Differ.

[113] Zhang S, Hong Z, Chai Y, Liu Z, Du Y, Li Q, Liu Q (2017). CSN5 promotes renal cell carcinoma metastasis and EMT by inhibiting ZEB1 degradation. Biochem Biophys Res Commun.

[114] Shen J, Hong L, Yu D, Cao T, Zhou Z, He S (2019). LncRNA XIST promotes pancreatic cancer migration, invasion and EMT by sponging miR-429 to modulate ZEB1 expression. Int J Biochem Cell Biol.

[115] Gao Y, Wang B, Luo H, Zhang Q, Xu M (2018). miR-217 represses TGF-β1-induced airway smooth muscle cell proliferation and migration through targeting ZEB1. Biomed Pharmacother.

[116] Zhou X, Men X, Zhao R, Han J, Fan Z, Wang Y, Lv Y, Zuo J, Zhao L, Sang M (2018). miR-200c inhibits TGF-β-induced-EMT to restore trastuzumab sensitivity by targeting ZEB1 and ZEB2 in gastric cancer. Cancer Gene Ther.

[117] Gu K, Li M-M, Shen J, Liu F, Cao J-Y, Jin S, Yu Y (2015). Interleukin-17-induced EMT promotes lung cancer cell migration and invasion via NF-κB/ZEB1 signal pathway. Am J Cancer Res.

[118] Liang W, Zhang Y, Zhou L, Lu X, Finn ME, Wang W, Shao H, Dean DC, Zhang L, Liu Y (2022). Zeb1 regulation of wound-healing-induced inflammation in alkali-damaged corneas. iScience.

[119] Sakuma Y (2017). Epithelial-to-mesenchymal transition and its role in EGFR-mutant lung adenocarcinoma and idiopathic pulmonary fibrosis. Pathol Int.

[120] Lamouille S, Xu J, Derynck R (2014). Molecular mechanisms of epithelial–mesenchymal transition. Nat Rev Mol Cell Biol.

[121] Tang LY, Heller M, Meng Z, Yu LR, Tang Y, Zhou M, Zhang YE (2017). Transforming growth factor-beta (TGF-beta) directly activates the JAK1-STAT3 axis to induce hepatic fibrosis in coordination with the SMAD pathway. J Biol Chem.

[122] Katsura A, Tamura Y, Hokari S, Harada M, Morikawa M, Sakurai T, Takahashi K, Mizutani A, Nishida J, Yokoyama Y, Morishita Y, Murakami T, Ehata S, Miyazono K, Koinuma D (2017). ZEB1-regulated inflammatory phenotype in breast cancer cells. Mol Oncol.

[123] Block CJ, Dyson G, Campeanu IJ, Watza D, Ratnam M, Wu G (2019). A stroma-corrected ZEB1 transcriptional signature is inversely associated with antitumor immune activity in breast cancer. Sci Rep.

[124] de Barrios O, Sanchez-Moral L, Cortés M, Ninfali C, Profitós-Pelejà N, Martínez-Campanario M, Siles L, Del Campo R, Fernández-Aceñero MJ, Darling DS (2019). ZEB1 promotes inflammation and progression towards inflammation-driven carcinoma through repression of the DNA repair glycosylase MPG in epithelial cells. Gut.

[125] Zhou ZY, Zhao WR, Zhang J, Chen XL, Tang JY (2019). Sodium tanshinone IIA sulfonate: a review of pharmacological activity and pharmacokinetics. Biomed Pharmacother.

[126] De Cock JM, Shibue T, Dongre A, Keckesova Z, Reinhardt F, Weinberg RA (2016). Inflammation triggers Zeb1-dependent escape from tumor latency. Cancer Res.

[127] Streit WJ, Mrak RE, Griffin WS (2004). Microglia and neuroinflammation: a pathological perspective. J Neuroinflamm.

[128] Colombo E, Farina C (2016). Astrocytes: key regulators of neuroinflammation. Trends Immunol.

[129] Li D, Lang W, Zhou C, Wu C, Zhang F, Liu Q, Yang S, Hao J (2018). Upregulation of microglial ZEB1 ameliorates brain damage after acute ischemic stroke. Cell Rep.

[130] Langfelder P, Cantle JP, Chatzopoulou D, Wang N, Gao F, Al-Ramahi I, Lu XH, Ramos EM, El-Zein K, Zhao Y, Deverasetty S, Tebbe A, Schaab C, Lavery DJ, Howland D, Kwak S, Botas J, Aaronson JS, Rosinski J, Coppola G, Horvath S, Yang XW (2016). Integrated genomics and proteomics define huntingtin CAG length-dependent networks in mice. Nat Neurosci.

[131] Weng R, Lu C, Liu X, Li G, Lan Y, Qiao J, Bai M, Wang Z, Guo X, Ye D (2018). Long noncoding RNA-1604 orchestrates neural differentiation through the miR-200c/ZEB axis. Stem Cells.

[132] Zhang T, Wu YC, Mullane P, Ji YJ, Liu H, He L, Arora A, Hwang HY, Alessi AF, Niaki AG, Periz G, Guo L, Wang H, Elkayam E, Joshua-Tor L, Myong S, Kim JK, Shorter J, Ong SE, Leung AKL, Wang J (2018). FUS regulates activity of microRNA-mediated gene silencing. Mol Cell.

[133] Shah PP, Lockwood WW, Saurabh K, Kurlawala Z, Shannon SP, Waigel S, Zacharias W, Beverly LJ (2015). Ubiquilin1 represses migration and epithelial-to-mesenchymal transition of human non-small cell lung cancer cells. Oncogene.

[134] Han B, Jiang W, Cui P, Zheng K, Dang C, Wang J, Li H, Chen L, Zhang R, Wang QM (2021). Microglial PGC-1α protects against ischemic brain injury by suppressing neuroinflammation. Genome Med.

[135] Bui T, Sequeira J, Wen TC, Sola A, Higashi Y, Kondoh H, Genetta T (2009). ZEB1 links p63 and p73 in a novel neuronal survival pathway rapidly induced in response to cortical ischemia. PLoS ONE.

[136] Yan XT, Lu JM, Wang Y, Cheng XL, He XH, Zheng WZ, Chen H, Wang YL (2018). XIST accelerates neuropathic pain progression through regulation of miR-150 and ZEB1 in CCI rat models. J Cell Physiol.

[137] Xu D, Ma X, Sun C, Han J, Zhou C, Chan MTV, Wu WKK (2021). Emerging roles of circular RNAs in neuropathic pain. Cell Prolif.

[138] Cai W, Zhang Y, Su Z (2020). ciRS-7 targeting miR-135a-5p promotes neuropathic pain in CCI rats via inflammation and autophagy. Gene.

[139] Siebzehnrubl FA, Silver DJ, Tugertimur B, Deleyrolle LP, Siebzehnrubl D, Sarkisian MR, Devers KG, Yachnis AT, Kupper MD, Neal D, Nabilsi NH, Kladde MP, Suslov O, Brabletz S, Brabletz T, Reynolds BA, Steindler DA (2013). The ZEB1 pathway links glioblastoma initiation, invasion and chemoresistance. EMBO Mol Med.

[140] Monteiro AR, Hill R, Pilkington GJ, Madureira PA (2017). The role of hypoxia in glioblastoma invasion. Cells.

[141] Maciaczyk D, Picard D, Zhao L, Koch K, Herrera-Rios D, Li G, Marquardt V, Pauck D, Hoerbelt T, Zhang W (2017). CBF1 is clinically prognostic and serves as a target to block cellular invasion and chemoresistance of EMT-like glioblastoma cells. Br J Cancer.

[142] Du X, Tu Y, Liu S, Zhao P, Bao Z, Li C, Li J, Pan M, Ji J (2020). LINC00511 contributes to glioblastoma tumorigenesis and epithelial–mesenchymal transition via LINC00511/miR-524-5p/YB1/ZEB1 positive feedback loop. J Cell Mol Med.

[143] Kahlert UD, Maciaczyk D, Doostkam S, Orr BA, Simons B, Bogiel T, Reithmeier T, Prinz M, Schubert J, Niedermann G (2012). Activation of canonical WNT/β-catenin signaling enhances in vitro motility of glioblastoma cells by activation of ZEB1 and other activators of epithelial-to-mesenchymal transition. Cancer Lett.

[144] Kowalski-Chauvel A, Gouaze-Andersson V, Baricault L, Martin E, Delmas C, Toulas C, Cohen-Jonathan-Moyal E, Seva C (2019). Alpha6-integrin regulates FGFR1 expression through the ZEB1/YAP1 transcription complex in glioblastoma stem cells resulting in enhanced proliferation and stemness. Cancers.

[145] Kowalski-Chauvel A, Modesto A, Gouaze-Andersson V, Baricault L, Gilhodes J, Delmas C, Lemarie A, Toulas C, Cohen-Jonathan-Moyal E, Seva C (2018). Alpha-6 integrin promotes radioresistance of glioblastoma by modulating DNA damage response and the transcription factor Zeb1. Cell Death Dis.

[146] Lim E-J, Kim S, Oh Y, Suh Y, Kaushik N, Lee J-H, Lee H-J, Kim M-J, Park M-J, Kim R-K (2020). Crosstalk between GBM cells and mesenchymal stemlike cells promotes the invasiveness of GBM through the C5a/p38/ZEB1 axis. Neuro Oncol.

[147] Shuttleworth SJ, Bailey SG, Townsend PA (2010). Histone deacetylase inhibitors: new promise in the treatment of immune and inflammatory diseases. Curr Drug Targets.

[148] Stridh P, ThessenHedreul M, Beyeen AD, Adzemovic MZ, Laaksonen H, Gillett A, Öckinger J, Marta M, Lassmann H, Becanovic K (2010). Fine-mapping resolves Eae23 into two QTLs and implicates ZEB1 as a candidate gene regulating experimental neuroinflammation in rat. PLoS ONE.

[149] George MF, Briggs FB, Shao X, Gianfrancesco MA, Kockum I, Harbo HF, Celius EG, Bos SD, Hedstrom A, Shen L, Bernstein A, Alfredsson L, Hillert J, Olsson T, Patsopoulos NA, De Jager PL, Oturai AB, Sondergaard HB, Sellebjerg F, Sorensen PS, Gomez R, Caillier SJ, Cree BA, Oksenberg JR, Hauser SL, D'Alfonso S, Leone MA, Martinelli Boneschi F, Sorosina M, van der Mei I, Taylor BV, Zhou Y, Schaefer C, Barcellos LF (2016). Multiple sclerosis risk loci and disease severity in 7,125 individuals from 10 studies. Neurol Genet.

[150] Jalaiei A, Asadi MR, Sabaie H, Dehghani H, Gharesouran J, Hussen BM, Taheri M, Ghafouri-Fard S, Rezazadeh M (2021). Long non-coding RNAs, novel offenders or guardians in multiple sclerosis: a scoping review. Front Immunol.

[151] Wendlandt EB, Graff JW, Gioannini TL, McCaffrey AP, Wilson ME (2012). The role of microRNAs miR-200b and miR-200c in TLR4 signaling and NF-κB activation. Innate Immun.

[152] Cha J-H, Kim K-W (2014). “Standby” EMT and “immune cell trapping” structure as novel mechanisms for limiting neuronal damage after CNS injury. Neural Regen Res.

[153] Zhao Q, Lu F, Su Q, Liu Z, Xia X, Yan Z, Zhou F, Qin R (2020). Knockdown of long noncoding RNA XIST mitigates the apoptosis and inflammatory injury of microglia cells after spinal cord injury through miR-27a/Smurf1 axis. Neurosci Lett.

[154] Gu S, Xie R, Liu X, Shou J, Gu W, Che X (2017). Long coding RNA XIST contributes to neuronal apoptosis through the downregulation of AKT phosphorylation and is negatively regulated by miR-494 in rat spinal cord injury. Int J Mol Sci.

[155] Poonaki E, Nickel A-C, ShafieeArdestani M, Rademacher L, Kaul M, Apartsin E, Meuth SG, Gorji A, Janiak C, Kahlert UD (2022). CD133-functionalized gold nanoparticles as a carrier platform for telaglenastat (CB-839) against tumor stem cells. Int J Mol Sci.

[156] Joseph JV, Conroy S, Pavlov K, Sontakke P, Tomar T, Eggens-Meijer E, Balasubramaniyan V, Wagemakers M, den Dunnen WF, Kruyt FA (2015). Hypoxia enhances migration and invasion in glioblastoma by promoting a mesenchymal shift mediated by the HIF1α–ZEB1 axis. Cancer Lett.

[157] Xia M, Hu M, Wang J, Xu Y, Chen X, Ma Y, Su L (2010). Identification of the role of Smad interacting protein 1 (SIP1) in glioma. J Neurooncol.

[158] Wang L, Liu Z, Balivada S, Shrestha T, Bossmann S, Pyle M, Pappan L, Shi J, Troyer D (2012). Interleukin-1β and transforming growth factor-β cooperate to induce neurosphere formation and increase tumorigenicity of adherent LN-229 glioma cells. Stem Cell Res Ther.

[159] Li Y, Wang L, Pappan L, Galliher-Beckley A, Shi J (2012). IL-1β promotes stemness and invasiveness of colon cancer cells through Zeb1 activation. Mol Cancer.

[160] Rosmaninho P, Mukusch S, Piscopo V, Teixeira V, Raposo AA, Warta R, Bennewitz R, Tang Y, Herold-Mende C, Stifani S, Momma S, Castro DS (2018). Zeb1 potentiates genome-wide gene transcription with Lef1 to promote glioblastoma cell invasion. EMBO J.

[161] Poonaki E, Ariakia F, Jalili-Nik M, ShafieeArdestani M, Tondro G, Samini F, Ghasemi S, Sahab-Negah S, Gorji A (2021). Targeting BMI-1 with PLGA–PEG nanoparticle-containing PTC209 modulates the behavior of human glioblastoma stem cells and cancer cells. Cancer Nanotechnol.

[162] Kurihara K, Isobe T, Yamamoto G, Tanaka Y, Katakura A, Tachikawa T (2015). Expression of BMI1 and ZEB1 in epithelial–mesenchymal transition of tongue squamous cell carcinoma. Oncol Rep.

[163] Lin T, Wang D, Chen J, Zhang Z, Zhao Y, Wu Z, Wang Y (2021). IL-24 inhibits the malignancy of human glioblastoma cells via destabilization of Zeb1. Biol Chem.

[164] Wang H, Jiang Z, Na M, Ge H, Tang C, Shen H, Lin Z (2017). PARK2 negatively regulates the metastasis and epithelial–mesenchymal transition of glioblastoma cells via ZEB1. Oncol Lett.

[165] Do H, Kim W (2018). Roles of oncogenic long non-coding RNAs in cancer development. Genom Inform.

[166] Rezaei O, Tamizkar KH, Sharifi G, Taheri M, Ghafouri-Fard S (2020). Emerging role of long non-coding RNAs in the pathobiology of glioblastoma. Front Oncol.

[167] Lv QL, Hu L, Chen SH, Sun B, Fu ML, Qin CZ, Qu Q, Wang GH, He CJ, Zhou HH (2016). A long noncoding RNA ZEB1-AS1 promotes tumorigenesis and predicts poor prognosis in glioma. Int J Mol Sci.

[168] Meng L, Ma P, Cai R, Guan Q, Wang M, Jin B (2018). Long noncoding RNA ZEB1-AS1 promotes the tumorigenesis of glioma cancer cells by modulating the miR-200c/141-ZEB1 axis. Am J Transl Res.

[169] Nesvick CL, Zhang C, Edwards NA, Montgomery BK, Lee M, Yang C, Wang H, Zhu D, Heiss JD, Merrill MJ (2016). ZEB1 expression is increased in IDH1-mutant lower-grade gliomas. J Neurooncol.

[170] Fratini L, Dalmolin MGS, Sinigaglia M, da Silveira Perla A, de Farias CB, Brunetto AL, Brunetto AT, da Cunha Jaeger M, Roesler R (2022). ZEB1 is a subgroup-specific marker of prognosis and potential drug target in medulloblastoma. NeuroMol Med.

[171] Muñoz-Hidalgo L, San-Miguel T, Megías J, Serna E, Calabuig-Fariñas S, Monleón D, Gil-Benso R, Cerdá-Nicolás M, López-Ginés C (2020). The status of EGFR modulates the effect of miRNA-200c on ZEB1 expression and cell migration in glioblastoma cells. Int J Mol Sci.

[172] Mao Y, Wei F, Wei C, Wei C (2018). microRNA-574 inhibits cell proliferation and invasion in glioblastoma multiforme by directly targeting zinc finger E-box-binding homeobox 1. Mol Med Rep.

[173] Chen W, Kong K-K, Xu X-K, Chen C, Li H, Wang F-Y, Peng X-F, Zhang Z, Li P, Li J-L (2018). Downregulation of miR-205 is associated with glioblastoma cell migration, invasion, and the epithelial–mesenchymal transition, by targeting ZEB1 via the Akt/mTOR signaling pathway. Int J Oncol.

[174] Song Y, Chen Y, Li Y, Lyu X, Cui J, Cheng Y, Zhao L, Zhao G (2018). Metformin inhibits TGF-β1-induced epithelial-to-mesenchymal transition-like process and stem-like properties in GBM via AKT/mTOR/ZEB1 pathway. Oncotarget.

[175] Pang H, Zheng Y, Zhao Y, Xiu X, Wang J (2015). miR-590-3p suppresses cancer cell migration, invasion and epithelial–mesenchymal transition in glioblastoma multiforme by targeting ZEB1 and ZEB2. Biochem Biophys Res Commun.

[176] Yue S, Wang L, Zhang H, Min Y, Lou Y, Sun H, Jiang Y, Zhang W, Liang A, Guo Y (2015). miR-139-5p suppresses cancer cell migration and invasion through targeting ZEB1 and ZEB2 in GBM. Tumor Biol.

[177] Troletti CD, de Goede P, Kamermans A, de Vries HE (1862). Molecular alterations of the blood–brain barrier under inflammatory conditions: the role of endothelial to mesenchymal transition. Biochim Biophys Acta (BBA) Mol Basis Dis.

[178] Leduc-Galindo D, Qvist P, Toth AE, Fryland T, Nielsen MS, Borglum AD, Christensen JH (2019). The effect of hypoxia on ZEB1 expression in a mimetic system of the blood-brain barrier. Microvasc Res.

[179] Arellano G, Acuña E, Reyes LI, Ottum PA, De Sarno P, Villarroel L, Ciampi E, Uribe-San Martín R, Cárcamo C, Naves R (2017). Th1 and Th17 cells and associated cytokines discriminate among clinically isolated syndrome and multiple sclerosis phenotypes. Front Immunol.

[180] Qian Y, Arellano G, Ifergan I, Lin J, Snowden C, Kim T, Thomas JJ, Law C, Guan T, Balabanov RD, Kaech SM, Miller SD, Choi J (2021). ZEB1 promotes pathogenic Th1 and Th17 cell differentiation in multiple sclerosis. Cell Rep.

[181] Benveniste EN, Liu Y, McFarland BC, Qin H (2014). Involvement of the janus kinase/signal transducer and activator of transcription signaling pathway in multiple sclerosis and the animal model of experimental autoimmune encephalomyelitis. J Interferon Cytokine Res.

[182] Smita S, Ahad A, Ghosh A, Biswas VK, Koga MM, Gupta B, Acha-Orbea H, Raghav SK (2018). Importance of EMT factor ZEB1 in cDC1 “MutuDC line” mediated induction of Th1 immune response. Front Immunol.

[183] Bao Y, Wang S, Xie Y, Jin K, Bai Y, Shan S (2018). MiR-28-5p relieves neuropathic pain by targeting Zeb1 in CCI rat models. J Cell Biochem.

[184] Zhang X, Zhang Y, Cai W, Liu Y, Liu H, Zhang Z, Su Z (2020). microRNA-128-3p alleviates neuropathic pain through targeting ZEB1. Neurosci Lett.

[185] Wang F, Wang L. MiR-96-5p relieves neuropathic pain by targeting ZEB1. 2020. 10.21203/rs.3.rs-124601/v1.

[186] Yan XT, Zhao Y, Cheng XL, He XH, Wang Y, Zheng WZ, Chen H, Wang YL (2018). Inhibition of miR-200b/miR-429 contributes to neuropathic pain development through targeting zinc finger E box binding protein-1. J Cell Physiol.

[187] Shen F, Zheng H, Zhou L, Li W, Zhang Y, Xu X (2019). LINC00657 expedites neuropathic pain development by modulating miR-136/ZEB1 axis in a rat model. J Cell Biochem.

[188] Chen YY, Jiang KS, Bai XH, Liu M, Lin SY, Xu T, Wei JY, Li D, Xiong YC, Xin WJ, Li ZY (2021). ZEB1 induces Ddr1 promoter hypermethylation and contributes to the chronic pain in spinal cord in rats following oxaliplatin treatment. Neurochem Res.

[189] Liu Y, Mo C, Mao X, Lu M, Xu L (2022). Increasing miR-126 can prevent brain injury after intracerebral hemorrhage in rats by regulating ZEB1. Contrast Media Mol Imaging.

[190] Vivinetto AL, Kim I-D, Goldberg DC, Fones L, Brown E, Tarabykin VS, Hill CE, Cho S, Cave JW (2020). Zeb2 is a regulator of astrogliosis and functional recovery after CNS injury. Cell Rep.

[191] Bundesen LQ, Scheel TA, Bregman BS, Kromer LF (2003). Ephrin-B2 and EphB2 regulation of astrocyte-meningeal fibroblast interactions in response to spinal cord lesions in adult rats. J Neurosci.

